# Protein Arginine Methyltransferase 1-mediated Histone H4R3 Dimethyl Asymmetric enhances Epidermal Growth Factor Receptor signaling to promote Peritoneal Fibrosis

**DOI:** 10.7150/ijbs.114037

**Published:** 2025-06-09

**Authors:** Hui Chen, Yingfeng Shi, Jinqing Li, Qin Zhong, Xiaoyan Ma, Yan Hu, Yishu Wang, Daofang Jiang, Xialin Li, Shasha Zhang, Shougang Zhuang, Na Liu

**Affiliations:** 1 Department of Nephrology, Shanghai East Hospital, Tongji University School of Medicine, Shanghai, China.; 2 Department of Medicine, Rhode Island Hospital and Alpert Medical School, Brown University, Providence, RI, USA.

**Keywords:** peritoneal fibrosis, protein arginine methyl transferase 1, asymmetric dimethylation of histone H4 on arginine 3, epidermal growth factor receptor

## Abstract

Peritoneal fibrosis (PF) is regarded as one of the major complications of peritoneal dialysis (PD) and is still lack of efficacious clinical interventions to address this issue. Previous publications have indicated that protein arginine methyltransferase 1 (PRMT1) is involved in the pathological process of various diseases. However, the role of PRMT1 in the development of PF remains to be elucidated. In the present study, we demonstrate that PRMT1 is highly expressed in the peritoneum and dialysis effluent of long-term PD patients, and that there is a positive correlation between PRMT1 and the hallmarks of fibrosis in human peritoneal specimens. Our results further demonstrate that the genetic depletion or pharmacological inhibition of PRMT1 has the potential to reduce extracellular matrix deposition and alleviate PF caused by high-glucose peritoneal dialysis fluid (HG-PDF) in murine models. In addition, silencing or pharmacological inhibition of PRMT1 could also reduce the epithelial-to-mesenchymal (EMT) phenotypic change caused by TGF-β1 *in vitro*. The use of RNA sequencing facilitated the identification of the epidermal growth factor receptor (EGFR) as a target of PRMT1 in PF. Furthermore, the reduction of PRMT1 levels, achieved through either genetic depletion or pharmacological inhibition, results in the deactivation of EGFR downstream signaling pathways. Our findings uncover a novel mechanism by which PRMT1-mediated H4R3me2a activates the EGFR and its associated downstream signaling pathways in the context of PF. Consequently, these findings imply that PRMT1 may serve as a valuable diagnostic or therapeutic target for PF.

## 1. Introduction

Peritoneal dialysis (PD) is a renal replacement therapy that is increasingly utilized for patients with end-stage kidney disease (ESKD). For instance, in 2020, the percentage of PD patients with ESKD in the United States was 12.7% 1. In comparison with hemodialysis, PD also has several advantages, including hemodynamic stability and greater schedule flexibility 2. The rising number of patients undergoing PD has prompted nephrologists to pay more attention to the complications and try to improve the quality of PD.

Peritoneal fibrosis (PF), one of the most serious complications of long-term PD, marked by loss of mesothelial cells with abnormal deposition of extracellular matrix (ECM) in sub-mesothelial areas 3-5. The development of PF results in the consequent loss of peritoneal integrity and transport function of the peritoneum, which may lead to ultrafiltration failure and PD drop-out 6. The mechanisms regulating PF have been widely studied. PF involves several processes, including the bio-incompatible dialysis fluid-induced chronic inflammation 7, angiogenesis in the peritoneum and epithelial-to-mesenchymal transition (EMT) 4, 8. The existing literature has reported various signaling pathways and cytokines that are involved in the following processes: transforming growth factor-β1(TGF-β1)/Smad signaling, signal transducer and activator of transcription (STAT), epidermal growth factor receptor (EGFR) signaling, protein kinase B (PKB1 or called serine/threonine kinase, AKT) signaling, extracellular signal-regulated kinase 1/2 (ERK1/2) signaling. It is important that other transcription factors and signaling pathways have been implicated in this process 4, 9, 10. Our previous study demonstrated that inhibition of EGFR attenuated high glucose-induced PF 11. However, considering the lack of effective therapy for PF in the clinic, further studies are still needed.

Several studies have revealed that post-translational modifications (PTMs) are important in the PF 10, 12. Arginine methylation, as one of the posttranslational modifications, has been shown to be closely associated with EMT and fibrosis 13, 14. The arginine methylation is catalyzed by Protein Arginine Methyltransferases (PRMTs), which exist extensively in mammalian cells 15, 16. The PRMT family comprises nine members, which can be classified into two types. PRMTs have the capacity to catalyze three types of methylation which regulate various biological processes, including monomethylation, asymmetrical dimethylation (ADMA) or symmetrical dimethylarginine (SDMA) 16-19. PRMT1 (a member of the type I PRMT) primarily catalyzes SDMA of histone H4 on arginine 3 (H4R3me2a), which has been shown to regulate pre-mRNA processing, protein stability, and numerous transcriptional activation pathways 16, 20. Recent studies have indicated the relationship between PRMT1 and fibrosis. For example, elevated expression of PRMT1 has been observed in the fibroblasts derived from idiopathic pulmonary fibrosis, and it has the ability to enhance IPF fibroblasts motility 21. In epicardial cells, PRMT1 has been demonstrated to control p53 stability, thereby regulating epicardial EMT 22. However, the role of PRMT1 in the progression of PF remains to be investigated in previous studies.

Thus, we investigated the role and mechanisms of PRMT1 in PF. In the current study, elevated levels of PRMT1 were observed in peritoneum specimens and dialysate from long-term peritoneal dialysis (PD) patients. PRMT1 served as a pro-fibrotic factor in PF as its positive correlation with the hallmarks of PF. In this study, we established murine PF model using high-glucose peritoneal dialysis fluid (HG-PDF) in PRMT1 conditional knockout mice and attempted to use AMI-1 to treat murine PF. Additionally, a cell injury model was established in human peritoneal mesothelial cells (HMrSV5). The generation of conditional PRMT1 knockout mice and the use of the PRMT1 inhibitor AMI-1 allowed us to confirm the function of PRMT1 in PF. Our findings indicate that both genetic and pharmacological inhibition of PRMT1 can alleviate PF *in vitro* and *in vivo*. In addition, RNA sequencing was employed to investigate the underlying mechanism by which PRMT1 regulates PF. The bioinformatic analysis suggested EGFR as the candidate target of PRMT1. We confirmed the relationship between PRMT1 and EGFR and found that PRMT1-mediated H4R3me2a could enhance the transcription of EGFR, and further activate EGFR-relevant regulatory pathways (including STAT3, AKT, and ERK1/2) and downstream targets such as Snail. The regulatory function of PRMT1 on EGFR would be inhibited by the genetic and pharmacological inhibition of PRMT1, indicating that PRMT1 may be an attractive therapeutic target for PF in the future.

## 2. Methods and Materials

All the other details for methods are in the Supplementary Methods.

### 2.1. Clinical samples collection and ethics statement

This study was approved by the Medical Ethics Committee of Shanghai East Hospital (2023-081) and conducted in accordance with the Declaration of Helsinki. Written informed consent was obtained from each patient.

For the experiments in the peritoneum samples of PD patients, 6 patients accepted catheter insertion operations, and 6 patients (duration of dialysis ≥ 26 months) accepted catheter removal operations because of ultrafiltration failure were enrolled in this study. All these patients underwent surgery at Shanghai East Hospital affiliated with Tongji University. These patients were divided into 2 groups (Table S1).

The PD effluent of patients with diverse PD duration time at 3 PD centers of Shanghai East Hospital, Shanghai Baoshan Hospital, and Shanghai Songjiang District Central Hospital. The included patients were divided into different 5 groups according to the PD duration: ≤ 1 month (n=16), 1-12 months (n=22), 12-24 months (n=13), 24-36 months (n=12), and > 36 months (n=17) (Table S2).

### 2.2. Mice

Animal experiments were reviewed and approved by the Institutional Animal Care and Use Committee at Tongji University (Shanghai, China). C57BL/6 mice were obtained from Shanghai Super-B&K Laboratory Animal Corp. Ltd, Shanghai, China. PRMT1^fl/fl^ mice were kindly provided by Prof. Shougang Zhuang's Lab. C57BL/6J Tg (Col1a2-Cre^+^/ERT2) mice (RRID: IMSR_JAX:029567 23) purchased from Jackson Lab (United States). Prmt1^fl/fl^ mice were bred with (Col1a2-Cre^+^/ERT2) mice to create inducible fibroblast-specific Prmt1-cKO mice (Prmt1^fl/fl^Col1a2-Cre^+^/ERT2, PRMT1 cKO mice). The gene deletion of Prmt1 in mice was induced by intraperitoneal injection of tamoxifen (Millipore Sigma, United States) at a dose of 75 mg/kg body weight for 5 consecutive days and wait 1 week to be used for further experiments. All mice were genotyped by PCR. All mice were on the C57BL/6J background and were maintained under specific pathogen-free conditions with a 12-hour light/ 12-hour dark cycle. Age-matched male mice were used for the experiments. Animal protocols involving animal care and use were approved by the Tongji University before initiation of any studies.

### 2.3. Murine PF model

To investigate the effects of the genetic depletion of PRMT1 in PF, we constructed PF murine model. The details of animals and treatment are provided in the Supplementary Methods.

### 2.4. Cell culture and treatments

Human peritoneal mesothelial cells (HMrSV5, purchased from Gino Biological Technology Co., Ltd., Guangzhou, Guangdong, PR China) were used in the current study. All of the *in vitro* experiments were repeated at least 3 times. The details of cell culture and treatments are provided in the Supplementary Methods.

### 2.5. siRNA transfection

The siRNAs for PRMT1 knockdown were purchased from Gene Pharma (Shanghai, China) and were transfected into the cells cultured in the antibiotic-free medium with lipofectamine 3000 (Invitrogen) according to the manufacturer's instructions. The sense 5'-3' of PRMT1 siRNA is CCAUCGACCUGGACUUCAATT and antisense 5'-3' is UUGAAGUCCAGGUCGAUGGTT. In parallel, scrambled siRNA (100 pmol) was used as the negative control (NC). After 24h transfection, the original antibiotic-free medium was changed, and cells were further treated with different types of treatment for another 36 hours. All the *in vitro* experiments were repeated at least three times.

## 3. Results

### 3.1. PRMT1 is highly expressed in the peritoneum specimens and dialysis effluent of long-term PD patients and positively correlates with fibrotic hallmarks

**Figure 1:**
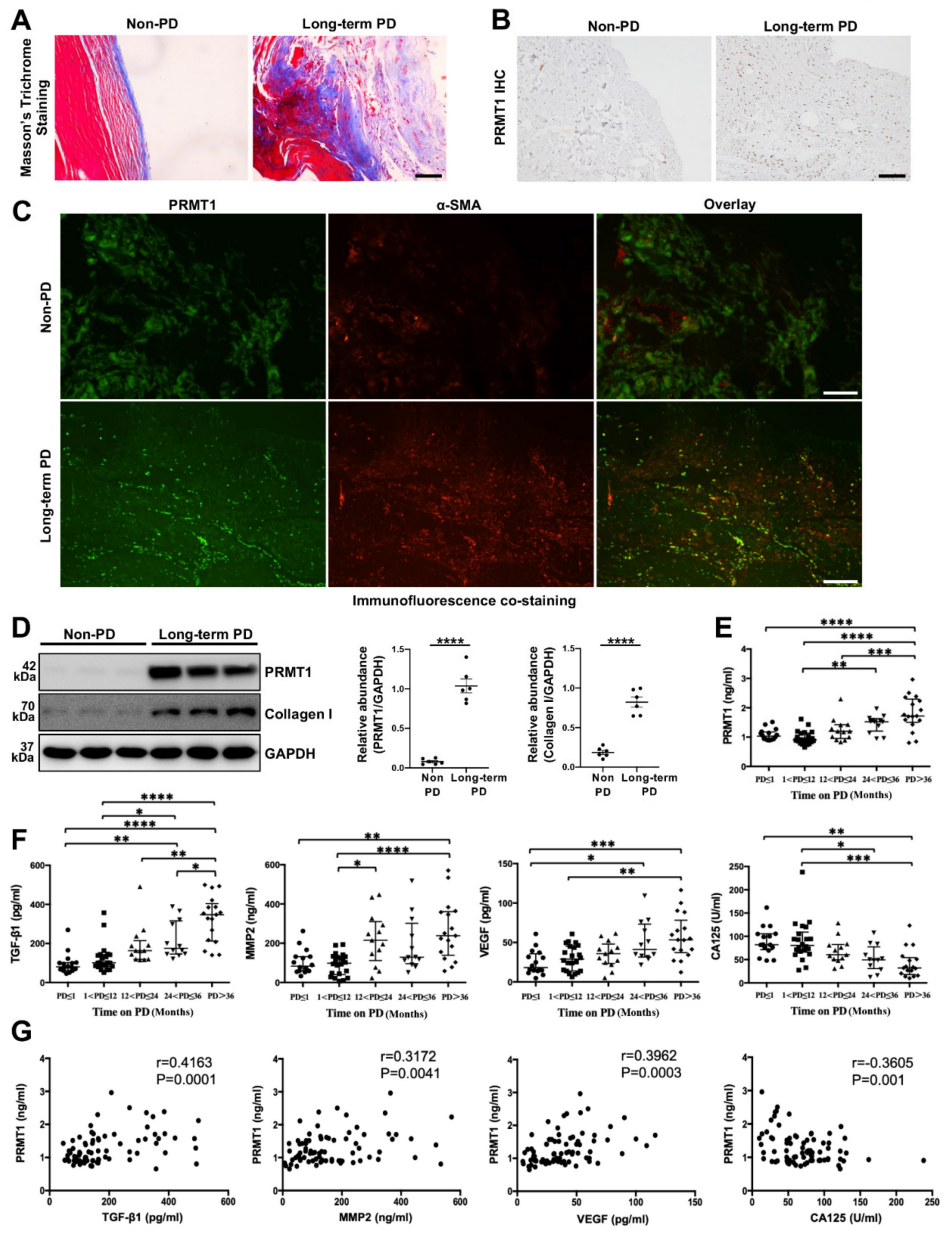
** PRMT1 overexpresses in the peritoneum of long-term PD patients.** For the experiments in the peritoneum samples of PD patients, 6 patients accepted catheter insertion operations, and 6 patients (duration of dialysis ≥ 26 months) accepted catheter removal operations because of ultrafiltration failure were enrolled in this study. **A)** Representative graphs of Masson's Trichrome staining in peritoneum samples from non-PD and long-term PD patients. Scale bars=50μm. **B)** Representative micrographs of immunostaining of PRMT1 in peritoneum from non-PD and long-term PD patients. Scale bars=100μm. **C)** Representative graphs of co-immunofluorescence staining of PRMT1 with α-SMA in the peritoneum of non-PD and long-term PD patients. Scale bars=100μm. **D)** The expression levels of PRMT1, Collagen Ⅰ and GAPDH were determined by western blot. The proteins were quantified by densitometry and normalized by GAPDH. **E)** The expression level of PRMT1 in the dialysis effluent of PD patients with different dialysis duration. **F)** The expression of TGF-β1, MMP2, VEGF, and CA125 in the dialysis effluent of PD patients with different dialysis duration. **G)** The correlation between the level of PRMT1 and level of TGF-β1, MMP2, VEGF, CA125 in the dialysis effluent. The included patients were divided into different 5 groups according to the PD duration: ≤ 1 month (n=16), 1-12 months (n=22), 12-24 months (n=13), 24-36 months (n=12), and > 36 months (n=17). Data were expressed as means ± SEM. N.S., no significant difference, *P<0.05, **P<0.01, ***P<0.001, ****P<0.0001.

Previous studies have demonstrated that PRMT1 is related to multiple fibrotic diseases 24, 25. Given this, we sought to investigate the potential role of PRMT1 in PF. The peritoneum specimens were obtained from non-PD patients and long-term PD patients. The peritoneum samples from Long-term PD patients who suffered from ultrafiltration failure had a more positive area in Masson's Trichrome Staining, showing a more severe fibrosis degree (Figure 1A). Next, we conducted the immunostaining of PRMT1 in the human peritoneum specimens and found the overexpression of PRMT1 in the fibrotic areas in the long-term PD patients' peritoneum (Figure 1B). The co-staining of PRMT1 and α-SMA (considered as one of the markers of the myofibroblasts and fibrosis 26, 27) showed that PRMT1 co-localized with α-SMA, suggesting a possible role of PRMT1 in fibroblasts activation in PF (Figure 1C). The western blotting also confirmed the high level of PRMT1 in the fibrotic tissues in long-term PD patients (Figure 1D). Meanwhile, the expression level of PRMT1 is positively correlated with the expression of Collagen I (Figure S1).

Several cytokines (such as TGF-β1, MMP2, VEGF, and CA125) in the PD effluent are reported to be capable to evaluate the condition of the peritoneal membrane 28. Consequently, we also detect these cytokines in the PD effluent. Further analysis of the level of PRMT1 in the dialysis effluent from PD patients with different dialysis durations revealed an upward trend in the level of PRMT1 as the time on PD increased (Figure 1E). In addition, we also detected the levels of TGF-β1, MMP2, VEGF, and CA125 in the dialysis effluent of patients (Figure 1F) and examined the relationship between PRMT1 and these hallmarks of PF (Figure 1G). The results demonstrated that PRMT1 positively correlated with TGF-β1, MMP2, and VEGF (which increased along with the PD time and promoted PF), while negatively correlated with CA125 (Figure 1G). Therefore, our data indicated that PRMT1 positively correlates with fibrotic hallmarks and may promote the progression of PF.

### 3.2. The genetic depletion of PRMT1 mitigates PF and reduces the ECM deposition in murine PF model

**Figure 2:**
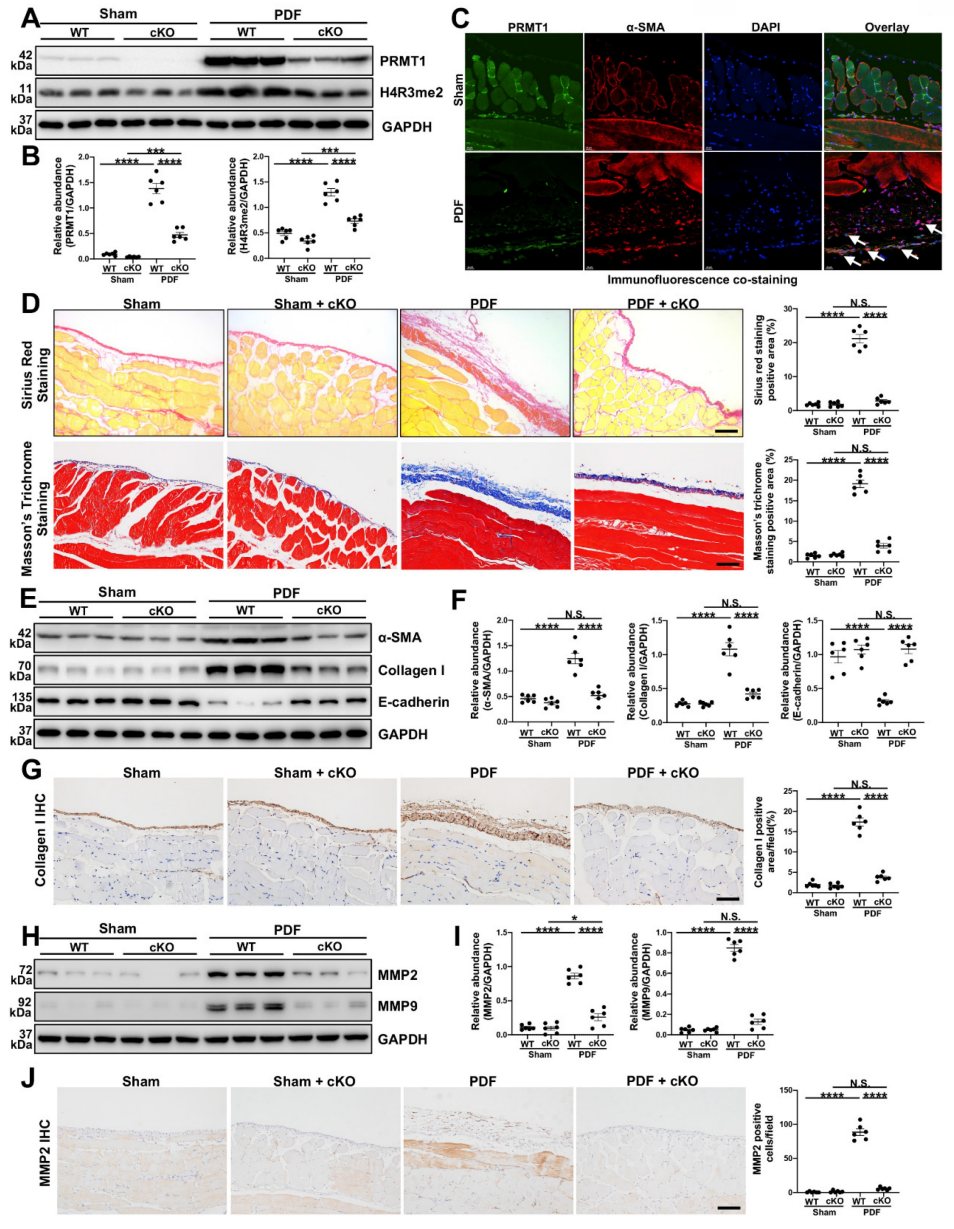
** Genetic depletion of PRMT1 alleviates PF caused by HG-PDF.** Prmt1^fl/fl^ mice were bred with (Col1a2-Cre^+^/ERT2) mice to create inducible fibroblast-specific Prmt1-cKO mice (Prmt1^fl/fl^Col1a2-Cre^+^/ERT2, PRMT1 cKO mice).** A)** The expression levels of PRMT1, H4R3me2 and GAPDH were determined by western blot, and **B)** the proteins were quantified by densitometry and normalized by GAPDH. **C)** Representative graphs of co-immunofluorescence staining of PRMT1 with α-SMA in the peritoneum of different groups of mice. Scale bars=100μm. **D)** Representative graphs of Masson's Trichrome staining (red signal represents muscle fibers and blood vessels, and blue signal represents collagen fibers) and Sirius Red Staining of the peritoneum of mice from different treatment groups. The positive area from each treatment group was quantified. Scale bars=100μm. **E)** The expression levels of α-SMA, Collagen Ⅰ, E-cadherin and GAPDH were determined by western blot, and **F)** the proteins were quantified by densitometry and normalized by GAPDH.** G)** Representative graphs of immunostaining of Collagen Ⅰ in mice peritoneum of different groups. The positive area from each treatment group was quantified. Scale bars=100μm. **H)** The expression levels of MMP2, MMP9 and GAPDH were determined by western blot, and **I)** the proteins were quantified by densitometry and normalized by GAPDH.** J)** Representative graphs of immunostaining of MMP2 in mice peritoneum of different groups. The positive area from each treatment group was quantified. Scale bars=100μm. Data were expressed as means ± SEM (n=6). N.S., no significant difference, *P<0.05, **P<0.01, ***P<0.001, ****P<0.0001.

As the global knockout of PRMT1 would lead to embryonic lethality 29, we tried to generate specific PRMT1 knockout mice. The immunofluorescence staining indicated the fibroblasts were the source of PRMT1 (Figure S2), thus we generate generated inducible fibroblast-conditional Prmt1-KO mice (PRMT1 cKO mice) (Figure S3). To substantiate the function of PRMT1 in PF, a mouse model of PF was then established. The PRMT1 cKO mice and wild-type mice (WT mice) were subjected to HG-PDF intraperitoneal injection. The protein level of PRMT1 and its major histone substrate H4R3me2a increased in the WT mice who received HG-PDF injection, while the levels in cKO mice significantly decreased (Figure 2A, B). Consistent with the results in human peritoneum specimens, PRMT1 co-localized in the α-SMA positive cells (Figure 2C). The morphic analysis showed that genetic depletion of PRMT1 could effectively alleviate the pathological lesions in mice peritoneum caused by HG-PDF (Figure 2D). The results indicated that PRMT1 genetic inhibition attenuated HG-PDF-induced fibrosis, evidenced by decreased levels of α-SMA, Collagen I, and increased levels of E-Cadherin (Fig 2E-G).

ECM deposition is the main histological change in peritoneal fibrosis. We next investigated the function of PRMT1 in regulating ECM in PF. The immunoblotting and immunostaining showed that the conditional knockout of PRMT1 could successfully suppress the ECM deposition (Figure 2H-J, Figure S4). Taken together, these results suggest that genetic depletion of PRMT1 in fibroblasts could alleviate the fibrosis and suppress the ECM deposition.

### 3.3. The pharmacological inhibition of PRMT1 alleviates the development of PF in murine PF model induced by HG-PDF

**Figure 3:**
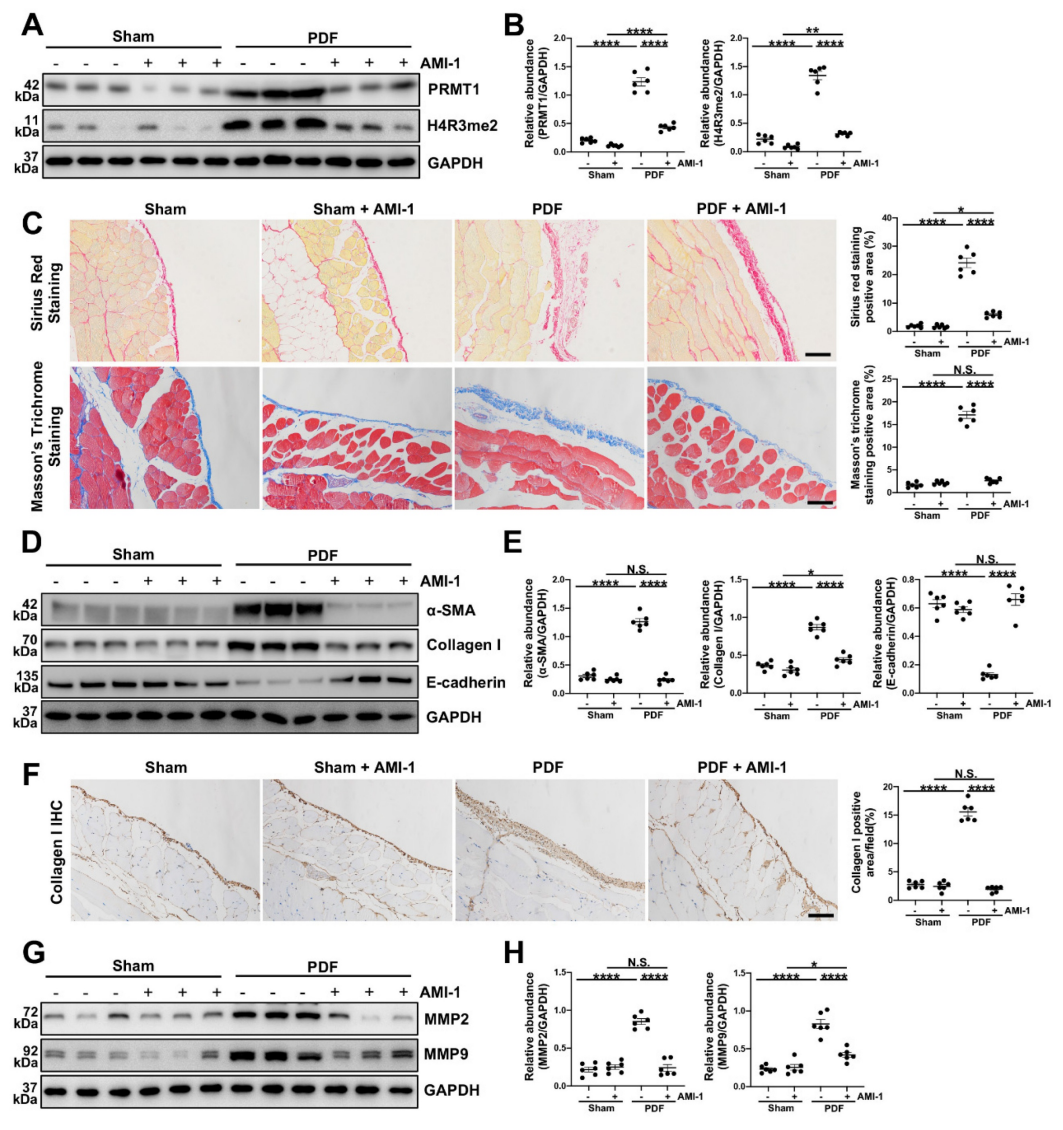
**Pharmacological inhibition of PRMT1 attenuates PF induced by HG-PDF.** To investigate the effects of pharmacological inhibition of PRMT1 in the PF, we used AMI-1 in mice.** A)** The expression levels of PRMT1, H4R3me2 and GAPDH were determined by western blot, and **B)** the proteins were quantified by densitometry and normalized by GAPDH. **C)** Representative graphs of Masson's Trichrome staining and Sirius Red Staining of the peritoneum of mice. The mice were treated with Vehicle or PRMT1 inhibitor AMI-1. The positive area from each treatment group was quantified. Scale bars=100μm. **D)** The expression levels of α-SMA, Collagen Ⅰ, E-cadherin and GAPDH were determined by western blot, and **E)** the proteins were quantified by densitometry and normalized by GAPDH.** F)** Representative graphs of immunostaining of Collagen Ⅰ in peritoneum of mice with different treatment. The positive area from each group was quantified. Scale bars=100μm. **G)** The expression levels of MMP2, MMP9 and GAPDH were determined by western blot, and **H)** the proteins were quantified by densitometry and normalized by GAPDH. Data were expressed as means ± SEM (n=6). N.S., no significant difference, *P<0.05, **P<0.01, ***P<0.001, ****P<0.0001.

To better confirm the function of PRMT1 in PF and explore its potential therapeutic value, we also examined the effects of pharmacological inhibition of PRMT1 on PF. AMI-1 is a PRMT1 methyltransferase inhibitor 30, and we showed that AMI-1 treatment effectively suppressed the expression of PRMT1 and H4R3me2a compared with the mice in PDF group only receiving vehicle treatment (Figure 3A, B). The administration of AMI-1 also successfully attenuated the pathological changes (Figure 3C) and the severity of fibrosis (Figure 3D-E). The immunostaining of Collagen Ⅰ also confirmed that the pharmacological inhibition of PRMT1 by AMI-1 could alleviate the fibrosis in murine model (Figure 3F). Furthermore, as what we found in the PRMT1 cKO mice, the injection of AMI-1 would also suppress the ECM deposition caused by HG-PDF (Figure 3G, H). The pharmacological inhibition of PRMT1, in a word, can alleviate the HG-PDF induced PF and suppress the ECM deposition in the peritoneum.

### 3.4. TGF-β1 can upregulate the PRMT1 expression, and the inhibition of PRMT1 abrogates the TGF-β1-induced phenotype changes *in vitro*

**Figure 4:**
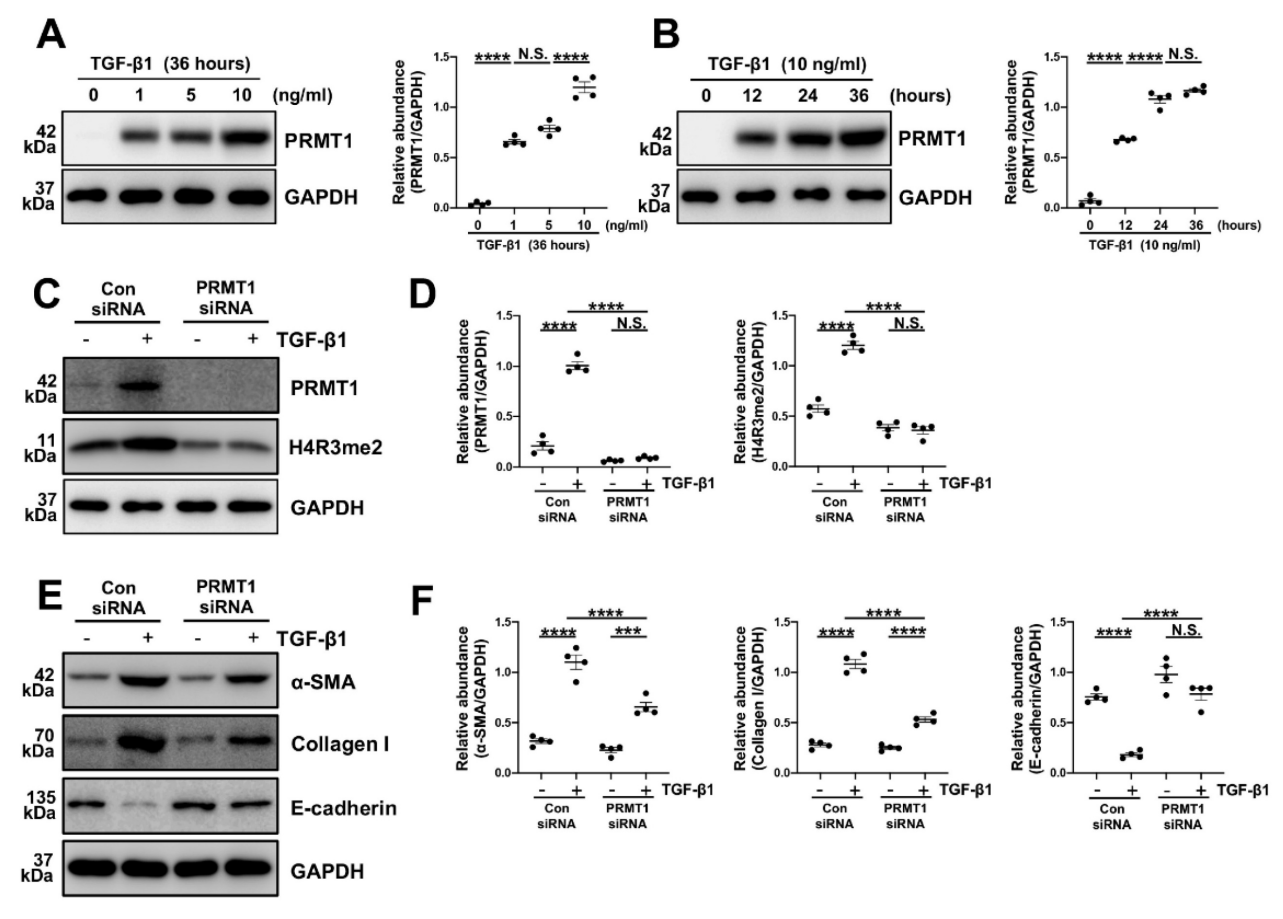
**TGF-β1 upregulates PRMT1 expression in HMrSV5, and inhibition of PRMT1 reduces EMT phenotype caused by TGF-β1 *in vitro*. A)** The expression levels of PRMT1 and GAPDH in the HMrSV5s treated with different doses of TGF-β1 were determined by western blot, and the proteins were quantified by densitometry and normalized by GAPDH. **B)** The expression levels of PRMT1 and GAPDH in the HMrSV5s treated with TGF-β1 for different time were determined by western blot, and the proteins were quantified by densitometry and normalized by GAPDH. **C)** The expression levels of PRMT1, H4R3me2 and GAPDH in HMrSV5s treated with PRMT1 siRNA or Negative Control (NC) in presence of TGF-β1 or Vehicle were determined by western blot, and **D)** the proteins were quantified by densitometry and normalized by GAPDH. **E)** The expression levels of α-SMA, Collagen I, E-cadherin and GAPDH in HMrSV5s with different treatments were determined by western blot, and **F)** the proteins were quantified by densitometry and normalized by GAPDH. Data were expressed as means ± SEM (n=4). N.S., no significant difference, *P<0.05, **P<0.01, ***P<0.001, ****P<0.0001.

Various studies have considered TGF-β1 as a key driving factor in PF 4, 31-33. As our findings in the dialysis effluents of PD patients indicated a positive correlation between TGF-β1 and PRMT1, we speculated that TGF-β1 may upregulate PRMT1 in the PF progression. To verify this hypothesis, the human peritoneal mesothelial cells (HMrSV5) were stimulated with TGF-β1. In HMrSV5s, we showed that TGF-β1 upregulated PRMT1 expression in a time-dependent and dose-dependent manner, as shown by increased protein level of PRMT1 (Figure 4A, B).

**Figure 5:**
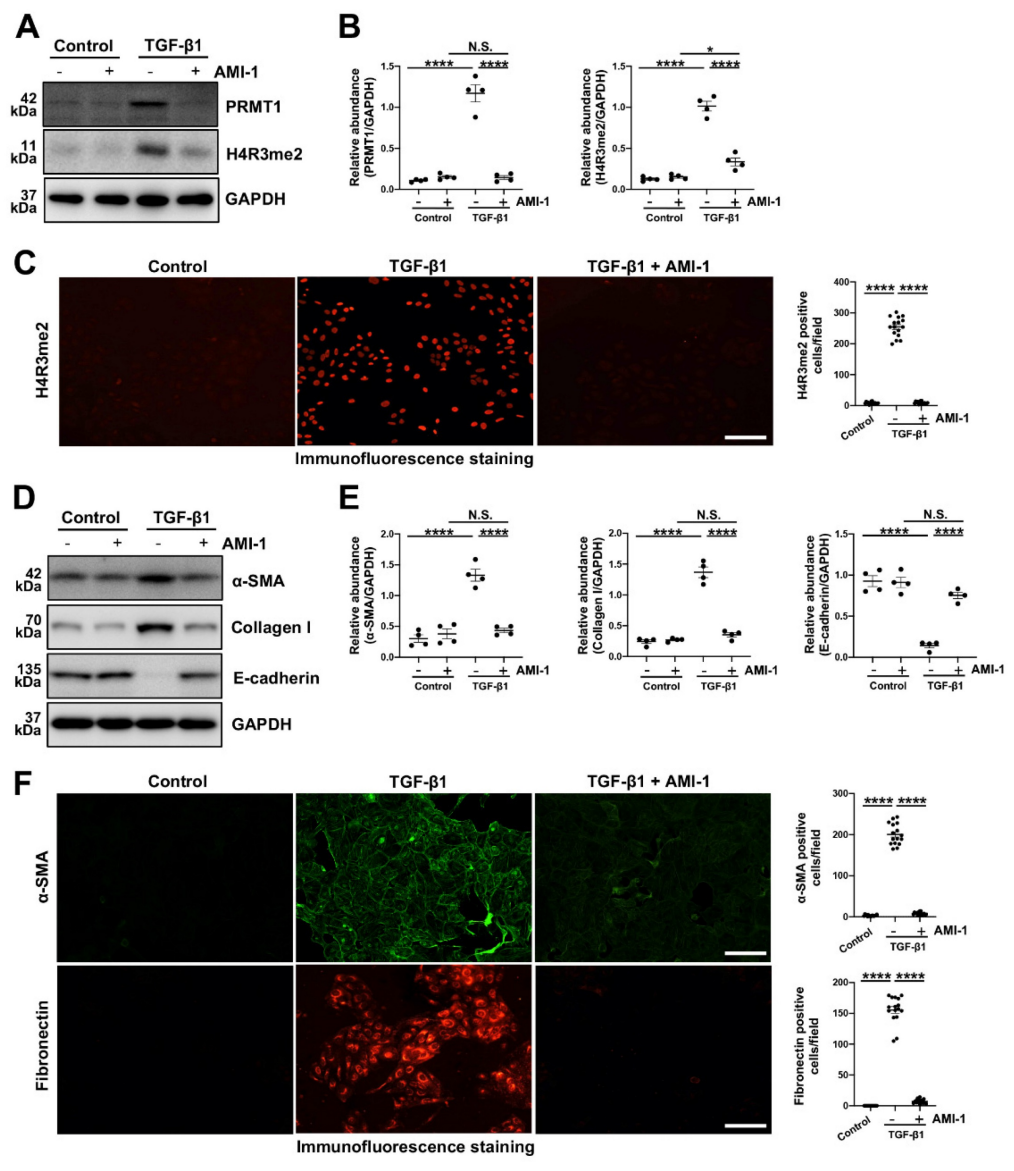
** Pharmacological inhibition of PRMT1 inhibits the EMT phenotype caused by TGF-β1 *in vitro*.** To verify the function and mechanisms of PRMT1 in PF, we used AMI-1 (5 μM) to treat HMrSV5 with stimulation of TGF-β1 (10 ng/ml) or vehicle for 36 hours.** A)** The expression levels of PRMT1, H4R3me2 and GAPDH in HMrSV5s pre-treated with AMI-1 or vehicle with or without the stimulation of TGF-β1 were determined by western blot, **B)** and the proteins were quantified by densitometry and normalized by GAPDH. **C)** Representative graphs of immunofluorescence staining of H4R3me2 in the HMrSV5s with different treatment. The count of positive cells in each high-power field (HPF) from each treatment group was quantified. Scale bars=100μm. **D)** The expression levels of α-SMA, Collagen Ⅰ, E-cadherin and GAPDH in TGF-β1-stimulated or vehicle-stimulated HMrSV5s with AMI-1 or vehicle were determined by western blot, and **E)** the proteins were quantified by densitometry and normalized by GAPDH. **F)** Representative graphs of immunofluorescence staining of α-SMA and Fibronectin in the HMrSV5s with different treatment. The count of positive cells in each HPF from each treatment group was quantified. Scale bars=100μm. Data were expressed as means ± SEM (n=4). N.S., no significant difference, *P<0.05, **P<0.01, ***P<0.001, ****P<0.0001.

The epithelial-to-mesenchymal transition (EMT) plays a huge part in the process of various fibrotic diseases and is one of the levers by which TGF-β1 regulates the fibrosis 3, 6, 34, 35. We then knocked down the PRMT1 in HMrSV5 by PRMT1 siRNA (Figure 4C, D). The genetic silencing of PRMT1 successfully inhibited the phenotype changes induced by TGF-β1 (characterized by a rise of α-SMA, Collagen I, and the drop of E-cadherin) (Figure 4E, F). Similarly, the pharmacological inhibition of PRMT1 in HMrSV5 by AMI-1 (Figure 5A-C) reduced the increases of α-SMA and Collagen I while rescued the decrease of E-cadherin caused by the TGF-β1 (Figure 5D, E). Accordingly, the immunofluorescence staining of α-SMA and Fibronectin confirmed the effects of AMI-1 to suppress TGF-β-induced EMT (Figure 5F). Taken together, these data suggest that TGF-β1 may upregulate the expression of PRMT1 in the progression of PF, and PRMT1 plays a crucial role in the TGF-β-induced phenotype changes *in vitro*.

### 3.5. PRMT1 catalyzes asymmetric dimethylation of histone H4 on arginine 3 to regulate EGFR

**Figure 6:**
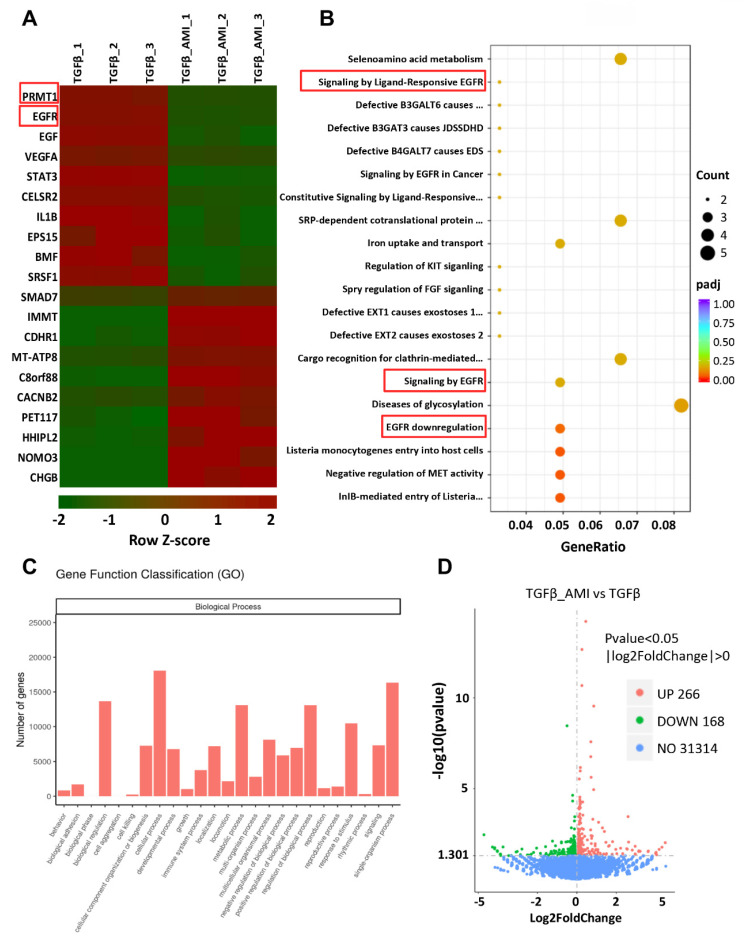
** Gene expression profile of AMI-1-treated human peritoneal mesothelial cells stimulated by TGF-β1 revealed by RNA sequencing analysis. A)** The heatmap showing the top differentially expressed genes (DEGs) between Vehicle and AMI-1-treated group HMrSV5 with TGF-β1 stimulation. Red signifies upregulated and green for downregulated genes. Differential gene expression was displayed as Z-score. **B)** Top 25 pathway enrichment result of GO biological process. **C)** Functional enrichment analysis of differentially expressed genes between different groups of HMrSV5s. **D)** A volcano plot showing 434 differentially expressed genes. There were 266 upregulated genes and 168 downregulated genes compared with the control group.

**Figure 7:**
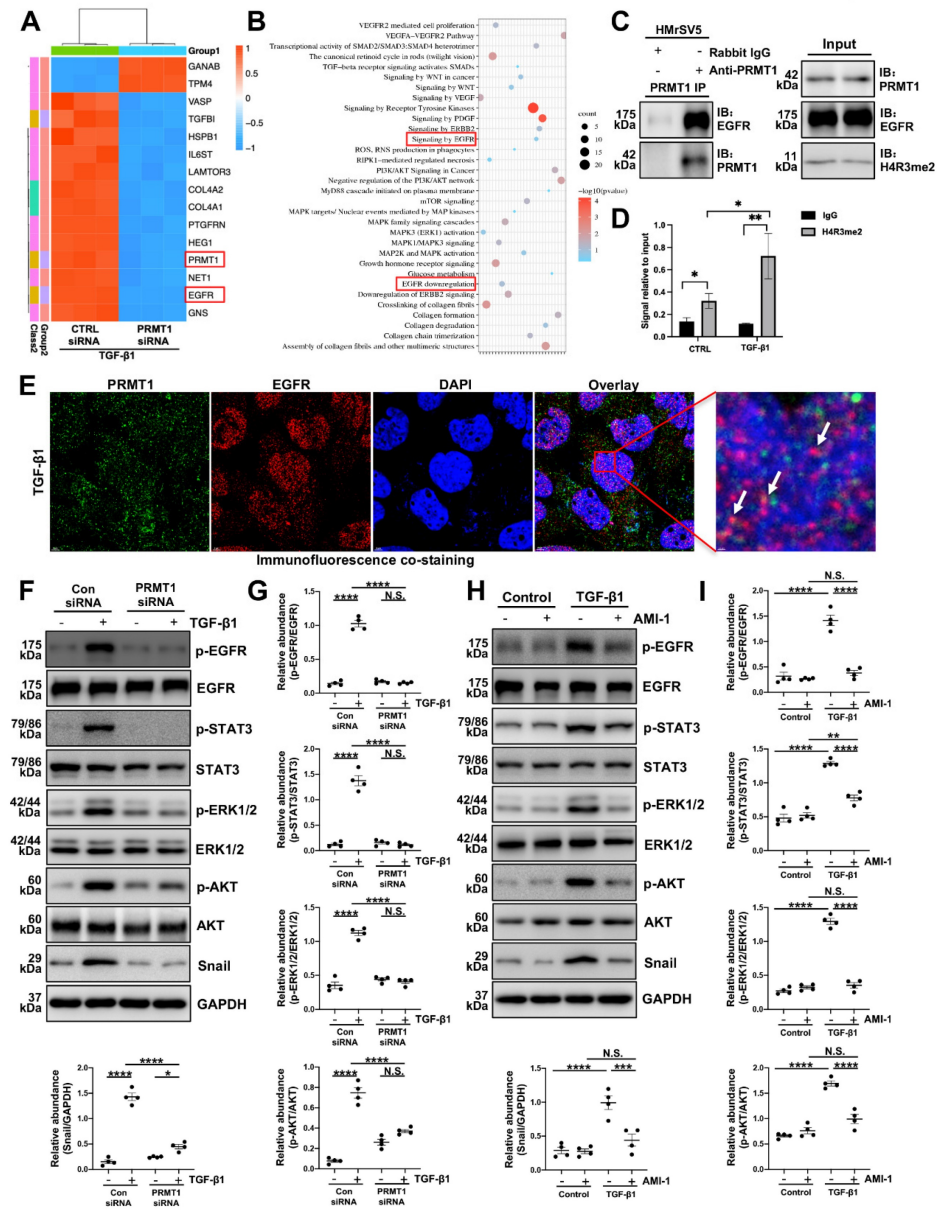
** PRMT1 mediates H4R3me2 to enhance EGFR translocation and further upregulate downstream signaling pathway. A)** The heatmap showing the top DEGs between PRMT1 knockdown and control group HMrSV5 with TGF-β1 stimulation. Red signifies upregulated and blue for downregulated genes. **B)** Gene ontology (GO) analysis showing the top enrichment pathways of DEGs. **C)** Association of PRMT1 with the EGFR. Whole-cell lysates were prepared and co-immunoprecipitation (co-IP) was performed with antibodies against the indicated proteins. The input was used as internal controls. **D)** CUT&RUN was carried out with IgG (negative control) and anti-H4R3me2 antibody. Q-PCR result for EGFR was shown as the percentage of input DNA. **E)** Representative images of co-immunofluorescence staining of PRMT1 with EGFR in the HMrSV5s stimulated by TGF-β1. Scale bars=3μm. **F)** The expressions of p-EGFR, EGFR, p-STAT3, STAT3, p-ERK1/2, ERK1/2, p-AKT, AKT, Snail and GAPDH in vehicle or TGF-β1-stimulated HMrSV5s transfected with NC or PRMT1 siRNA were determined by western blot, and the proteins **G)** were quantified by densitometry and normalized. GAPDH served as a loading control.** H)** The expressions of p-EGFR, EGFR, p-STAT3, STAT3, p-ERK1/2, ERK1/2, p-AKT, AKT, Snail and GAPDH in vehicle or TGF-β1-stimulated HMrSV5s treated with vehicle or AMI-1 were determined by western blot, and the proteins **I)** were quantified by densitometry and normalized. GAPDH served as a loading control. Data were expressed as means ± SEM (n=4). N.S., no significant difference, *P<0.05, **P<0.01, ***P<0.001, ****P<0.0001.

To elucidate the molecular mechanism of PRMT1 in PF, first, we conducted bulk RNA sequencing on TGF-β-stimulated HMrSV5 cells treated with AMI-1 or vehicle. The heatmap revealed distinct differences in gene expression between the two groups (Figure 6A). Bioinformatic analysis identified 434 differentially expressed genes (DEGs) (with 266 up-regulated and 168 down-regulated), and we further performed the pathways and functions enrichment analysis (Figure 6B-D). Among the top DEGs and the processes (according to GO annotations) in which the DEGs were involved, EGFR and its related signaling pathway aroused our interest, especially in biological regulation, cellular process metabolic process, etc. Then, we procedure bulk RNA-seq on TGF-β-stimulated HMrSV5 cells with or without PRMT1 knockdown by siRNA also identified epidermal growth factor receptor (EGFR) as one candidate target gene of PRMT1 (Figure 7A, B and Figure S5). From the DEGs identified in the transcriptome analysis, in response to TGF-β1 stimulation, 1602 genes were up-regulated, and 1446 genes were significantly down-regulated. Also, with gene knockdown of PRMT1, by Venn diagram analysis, among the differential genes up-regulated due to TGF-β1 stimulation, 68 genes were suppressed because of knockdown of PRMT1 expression, such as TGF-β1, EGFR, COL4A1, and so on (Figure S6A). We then constructed an interaction network integrating EGFR with other identified DEGs from the RNA sequencing to identify key molecules interacting with EGFR. The protein-protein interaction (PPI) network analysis (Figure S6B) recognized the TGF-β1 and EGFR as the hub genes.

Next, the immunoprecipitation confirmed the interaction between PRMT1 and EGFR, suggesting that PRMT1 may physically interact with EGFR in HMrSV5 cells (Figure 7C). Cleavage Under Targets & Release Using Nuclease (CUT&RUN) assay results further demonstrated that PRMT1 directly regulated EGFR by its function to catalyze asymmetric dimethylation of histone H4 on arginine 3 (Figure 7D). The co-staining of PRMT1 with EGFR in the TGF-β-stimulated cells indicates the enhancement function of PRMT1 on EGFR (Figure 7E). Collectively, the results of RNA sequencing and our experiments indicate the regulatory function of PRMT1 on EGFR.

### 3.6. Genetic or pharmacological inhibition of PRMT1 suppresses the activation of EGFR and its downstream pathways *in vitro*

EGFR can activate subsequent signaling pathways and participate in various of biological processes 36. To verify the involvement of PRMT1 in regulating EGFR and its downstream signaling, we treated HMrSV5 cells with TGF-β1 and investigated the expression of PRMT1 and the downstream targets. TGF-β significantly made the levels of phospho-EGFR (p-EGFR), phospho-STAT3 (p-STAT3), phospho-ERK1/2 (p-EGFR), phospho-AKT (p-AKT) and Snail elevate (Figure 7F-I). However, genetic deletion of PRMT1 significantly suppressed the TGF-β-induced EGFR-STAT3 signal activation, phosphorylation of the downstream ERK1/2, AKT, and elevation of Snail (Figure 7F, G). The pharmacological inhibition of PRMT1 by AMI-1 also has similar results (Figure 7H, I). AMI-1 suppressed the activation of EGFR and its downstream targets in the HMrSV5 cells (Figure 7H, I). Furthermore, the staining of Snail confirmed that the genetic and pharmacological inhibition of PRMT1 could suppress the upregulation of downstream targets of EGFR (Figure S7-8).

### 3.7. Genetic or pharmacological inhibition of PRMT1 abrogates the activation of EGFR and its downstream pathways *in vivo*

**Figure 8:**
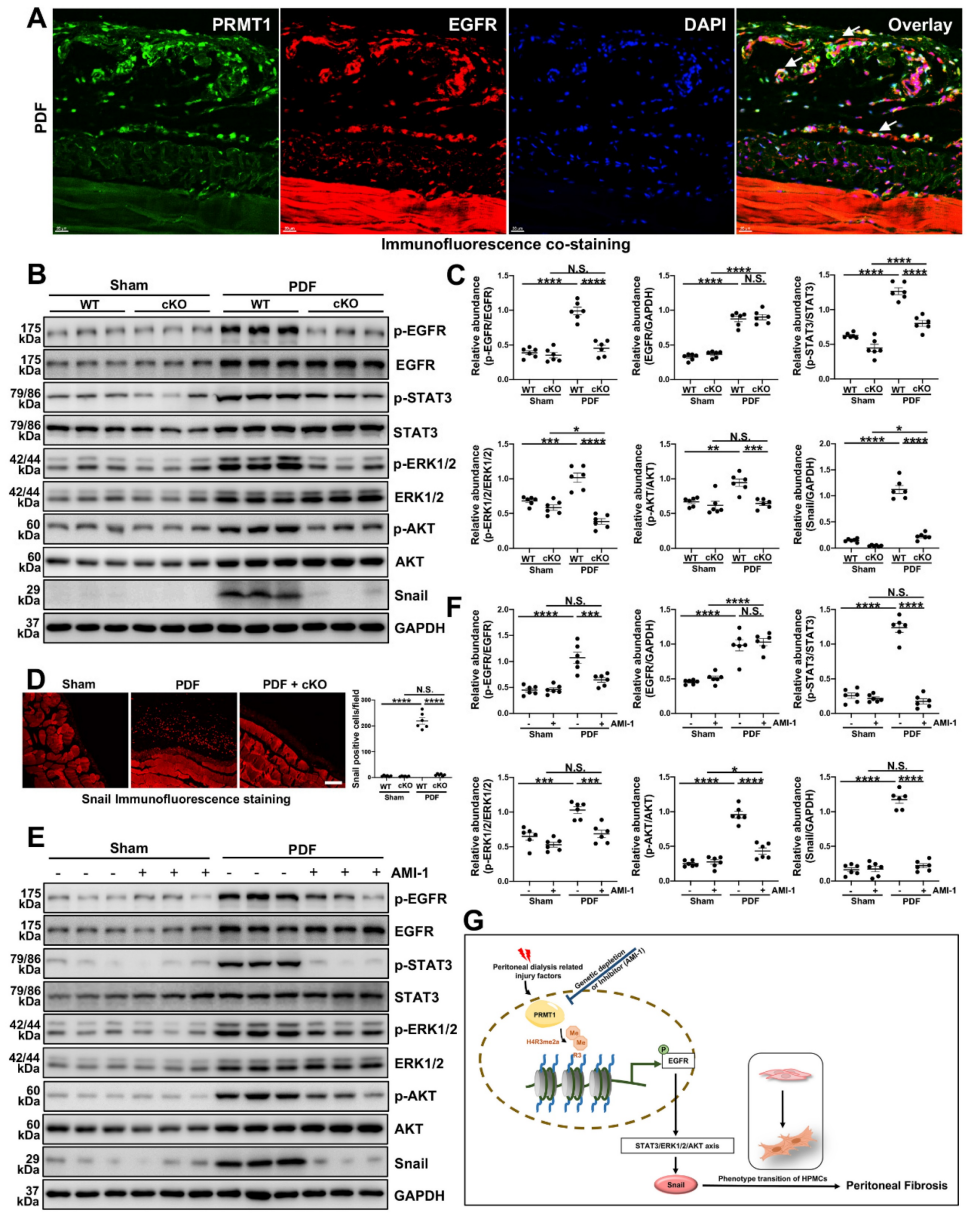
** PRMT1 mediates H4R3me2 to enhance EGFR translocation and further activate its downstream signaling pathway. A)** Representative images of co-immunofluorescence staining of PRMT1 with EGFR in the peritoneum of mice received HG-PDF intraperitoneal injection for 28 days. Scale bars=20μm. **B)** The expressions of p-EGFR, EGFR, p-STAT3, STAT3, p-ERK1/2, ERK1/2, p-AKT, AKT, Snail and GAPDH in the peritoneum tissue samples of wildtype or PRMT1-cKO mice injected with vehicle or HG-PDF for 28 days were determined by western blot, and the proteins **C)** were quantified by densitometry and normalized. GAPDH served as a loading control.** D)** Representative graphs of immunofluorescence staining of Snail in the peritoneum of mice received 28 days-long-HG-PDF intraperitoneal injection. The positive cell from each group was quantified. Scale bars=100μm. **E)** The expressions of p-EGFR, EGFR, p-STAT3, STAT3, p-ERK1/2, ERK1/2, p-AKT, AKT, Snail and GAPDH in in the peritoneum tissue samples of mice from different treatment groups were determined by western blot, and the proteins **F)** were quantified by densitometry and normalized. GAPDH served as a loading control. **G)** Schematic working model for the mechanisms by which PRMT1 mediates H4R3me2 enhancing EGFR signaling to promote peritoneal fibrosis. Data were expressed as means ± SEM (n=6). N.S., no significant difference, *P<0.05, **P<0.01, ***P<0.001, ****P<0.0001.

In addition to the *in vitro* experiments, we also evaluated the effects of inhibition of PRMT1 on EGFR and the related signaling. Similar to the findings in HMrSV5 cells, the co-staining of PRMT1 with EGFR in the mice peritoneum showed that HG-PDF induced the high expression and co-localization of EGFR and PRMT1 (Figure 8A). The Western blotting verified the results of staining. The HG-PDF injection induced the activation of EGFR and the downstream STAT3/ERK1/2/AKT signaling, characterized by increased phosphorylation levels (Figure 8B, C). The immunofluorescence staining showed the upregulation of Snail in the mice peritoneum in the WT+PDF group (Figure 8D). However, the upregulation of EGFR and activation of the EGFR-related signaling pathways were suppressed in the PRMT1-cKO mice (Figure 8B-D). Likewise, the AIM-1 treatment also inhibited the phosphorylation of EGFR, STAT3, ERK1/2, and AKT (Figure 8E, F). These data suggest whether genetic deletion or pharmacological inhibition of PRMT1 could suppress the activation of EGFR and the downstream STAT3/ERK1/2/AKT signaling.

## 4. Discussion

The long-standing lack of effective therapy for PF necessitates conducting in-depth studies of the molecular mechanisms of PF and the identification of novel therapeutic targets. While accumulating data indicate the importance of arginine methylation in fibrotic diseases, the role of arginine methyltransferase in PF remains unreported. In the current study, we demonstrate that inhibition of PRMT1 alleviates peritoneal fibrosis by inhibiting the demethylation of H4R3 thereby suppressing the activation of EGFR and downstream signaling pathways. We summarized the mechanisms by which PRMT1 regulates PF in **Figure 8G**: PD-related injury factors, such as bioincompatible PD solutions, produce glycation end products that activate TGF-β and further upregulate PRMT1. The up-regulated PRMT1 then mediates H4R3me2a, which enhances the expression and activation of EGFR. EGFR activation directly triggers activation of the downstream STAT3, AKT, and ERK1/2 signaling pathways, leading to the upregulation of the transcription factor Snail. Activating the above-mentioned pathways results in the phenotypic transition of human peritoneal mesothelial cells and the promotion of PF.

Our findings have significant clinical implications. Initially, we identified the overexpression of PRMT1 in the peritoneum specimens and dialysis effluents from long-term dialysis patients. Our data revealed that the level of PRMT1 in the dialysis fluid increased as the duration of dialysis increased. The ELISA assays also exhibited a consistent increasing trend in PRMT1 levels with the trend of levels of TGF-β1, MMP2, VEGF, and an inverse correlation with the trend of CA125 in dialysis effluent. These cytokines have been linked to fibrogenesis, tissue remodeling, angiogenesis and the loss of mesothelial cells 37, 38. Especially for CA125, it has high clinical guidance value for PD patients. CA125 is a glycoprotein secreted by peritoneal mesothelial cells into the dialysis effluent, and its concentration reflects the viability and mass of the mesothelial cell layer 28, 39, 40. A decline in CA125 levels over time may indicate peritoneal membrane deterioration, such as peritoneal fibrosis and angiogenesis induced by long-term exposure to PD solutions 28, 39, 40. In our study, we found that PRMT1 was negatively correlated with CA125 according to the ELISA results, which suggests the possibility of PRMT1 to be a novel predictive indicator of PF and peritoneal function deterioration.

Recently, PRMT1 has indicated that PRM1 may have oncogenic function in tumor cells 18. A study suggested that PRMT1 overexpression may inhibit differentiation and contribute to the development of blood cancers 41. Furthermore, the importance of PRMT1 in the cardiac system has also been highlighted. The loss of PRMT1 in the cardiomyocyte would result in a disruption of homeostasis and lead to heart failure 42. In addition, PRMT1 has been proven to be associated with pulmonary fibrosis. In the lungs of patients with idiopathic pulmonary fibrosis and mice treated with bleomycin, the PRMT1 expression is elevated 21. The knockout of PRMT1 in the hepatic stellate cells in mice alleviates liver fibrosis by suppressing NF-κB and TGF-β signaling 24. However, the role of PRMT1 in peritoneal fibrosis has never been reported before. In this study, we generated the inducible fibroblast-specific PRMT1-cKO mice to investigate the role of PRMT1. The deletion of PRMT1 substantially mitigated pathological lesions and ECM deposition in the peritoneum induced by HG-PDF. The administration of the PRMT1 inhibitor AMI-1 achieved similar results. *In vitro*, we observed that TGF-β could upregulate the expression of PRMT1. The genetic silencing and pharmacological inhibition of PRMT1 effectively suppress the phenotypic changes induced by TGF-β, which is recognized as a key factor in PF development 3-5, 10. The results are in accordance with the previous publications. It is reported that the interaction between TGF-β1 and arginase-1 contributes to lung fibrosis in mice 43. In the human epithelial cells, PRMT1 methylates SMAD7 to facilitate TGF-β1-induced EMT and proliferation 44. Another research in ovarian cancer found that PRMT1-mediated BRD4-ADMA modification is associated with TGF-β signaling and promotes metastasis and invasion 45. Nevertheless, the precise way TGF-β1 interacts with PRMT1 in PF requires further investigation.

In addition, we identified EGFR as a potential target of PRMT1 in the progression of PF through transcriptomic analysis. The RNA-seq results also confirmed that PRMT1 inhibition in the human peritoneal mesothelial cells markedly decreased the EGFR expression with relatively decreased activation of the downstream signaling pathways. EGFR belongs to the family of the ErbB tyrosine kinase receptors 36, 46, 47. The epidermal growth factor (EGF) induces the EMT through phosphorylation of EGFR, which subsequently activates the subsequent signaling pathways, resulting in MMP2- and MMP9-mediated proteolysis of ECM depending on the ERK MAPK pathway 36, 48, 49. Currently, studies have reported common downstream targets of the EGFR pathway, including STAT3, AKT, ERK, and Snail, which play important roles in cellular proliferation, differentiation, and survival 36, 46-49. Besides being overexpressed in many types of cancer, EGFR is also associated with fibrosis 50, 51. Phosphorylated EGFR and p-AKT are apparently elevated in the myofibroblasts of idiopathic pulmonary fibrosis patients 52. The inhibition of MEK/ERK prevents the progression of pulmonary fibrosis 53. In rats with PF, inhibiting core fucosylation of EGFR could suppress phosphorylation of STAT3 and alleviate PF 54. We also previously identified the protective effect of inhibition of EGFR in the development and progression of PF 11, 55. Based on the results of RNA sequencing and bioinformatics analysis, we speculated that EGFR signaling and its downstream pathways are the main mechanism of PRMT1 regulation of PF. Our experiment results indicate the interaction between PRMT1 and EGFR. The CUT&RUN assays suggest that PRMT1-mediate H4R3me2a regulates the expression and activation of EGFR. Our data demonstrated that PRMT1 blockade downregulated the expression of EGFR in mice peritoneum and cells, along with inhibition of the downstream signaling and transcription factor Snail. These results agree with the previous studies. *Nakai K et al.* have reported the significance of PRT1 to the activity of EGFR in the triple-negative-breast cells 56. A previous study has demonstrated that the methylations on the EGFR extracellular domain mediated by PRMT1 enhance receptor function in colorectal cancer cells, affecting the EGF-EGFR binding affinity to promote tumorigenesis 57. In colorectal cancer, PRMT1 cooperates with chromatin subfamily A member 4 (SMARCA4) to activate EGFR signaling, promoting malignant transitions 58. Overall, regulatory effects of the PRMT1 on EGFR in PF warrant detailed investigation.

Therefore, we believe that PRMT1 promotes peritoneal fibrosis through the regulation of EGFR, but EGFR may not be its only substrate protein during the process of peritoneal fibrosis. In recent research, PRMT1 was reported to mediate BRD4 arginine methylation and phosphorylation thus promoting partial epithelial-mesenchymal transformation and renal fibrosis 59. Another study confirmed PRMT1 as an essential mediator of TGF-β signaling that regulated the epithelial-mesenchymal transition and epithelial cell stemness through SMAD7 methylation 44. PRMT1 may also exert its effect through the substrate proteins mentioned above in peritoneal fibrosis. In addition, we have also discovered other fibrosis-related proteins after PRMT1 inhibition according to the RNA sequencing. And we will confirm other substrate proteins in future experiments.

Moreover, the pharmacological inhibition of PRMT1 by AMI-1 in this study showed satisfying therapeutic effects on PF, thus providing a potential therapy for PF. AMI-1 is a pan-type I PRMT inhibitor and could ameliorate inflammation, collagen deposition, and fibrosis in the pulmonary 58. However, the selectivity of AMI-1 is not specific. Previous studies indicated that AMI-1 could also inhibit the activity of PRMT5 60. Further researches would be required to ascertain the efficacy of PRMT1-specific inhibitors in the treatment of PF.

In conclusion, the present study highlights the role of PRMT1 in PF and originally reveals that PRMT1 promotes PF by mediating H4R3me2a to enhance the expression of EGFR and activate the EGFR, STAT3, AKT, and ERK signaling. The present study contributes to the advancement of the understanding of the mechanisms underlying PF and provides novel insights that PRMT1 may be a novel predictive factor and therapeutic target of PF, thus providing a brand-new therapeutic strategy for PF.

## Supplementary Material

Supplementary materials and methods, figures and tables.

## Abbreviations

ADMA: Asymmetrical Dimethylation
AKT: Serine/Threonine Kinase
CA125: Cancer Antigen 125
cKO: Conditional Knock-out
ECM: Extracellular matrix
EGF: epidermal growth factor
EGFR: Epidermal Growth Factor Receptor
EMT: Epithelial-to-mesenchymal
ERK1/2: Extracellular Signal-regulated Kinase 1/2
ESKD: End-stage kidney disease
H4R3me2a: SDMA of histone H4 on arginine 3
HG-PDF: High-glucose peritoneal dialysis fluid
MMP2: Matrix Metalloproteinase 2
NF-κB: Nuclear factor-κB
PD: Peritoneal Dialysis
PF: Peritoneal Fibrosis
PRMT1: Protein Arginine Methyltransferase
PRMTs: Protein arginine methyltransferases
PTM: Post-translational Modifications
SDMA: Symmetrical Dimethylarginine
STAT: Smad signaling, signal transducer and activator of transcription
TGF-β1: Transforming Growth Factor-β1
VEGF: Vascular Endothelial Growth Factor
α-SMA: α-smooth muscle actin
