# Supplementary material for: Protein Arginine Methyltransferase 1-mediated Histone H4R3 Dimethyl Asymmetric enhances Epidermal Growth Factor Receptor signaling to promote Peritoneal Fibrosis
Source: Int J Biol Sci. 2025 Jun 9;21(9):4010–26. doi: 10.7150/ijbs.114037 (PMC12223772; doi:10.7150/ijbs.114037)
Supplement: Supplementary file 1 — Supplementary materials and methods, figures and tables. [file ijbsv21p4010s1.pdf]

Protein Arginine Methyltransferase 1-mediated Histone H4R3 Dimethyl Asymmetric enhances Epidermal Growth Factor Receptor signaling to promote Peritoneal Fibrosis

Hui Chen[1*], Yingfeng Shi[1*], Jinqing Li[1*], Qin Zhong[1], Xiaoyan Ma[1], Yan Hu[1], Yishu Wang[1], Daofang Jiang[1], Xialin Li[1], Shasha Zhang[1], Shougang Zhuang[1,2], Na Liu[1]

[1]Department of Nephrology, Shanghai East Hospital, Tongji University School of Medicine, Shanghai, China;

[2]Department of Medicine, Rhode Island Hospital and Alpert Medical School, Brown University, Providence, RI, USA.

* Hui Chen, Yingfeng Shi and Jinqing Li contributed equally to this work.

Correspondence and offprint requests to: Na Liu, M.D., Ph.D., Department of Nephrology, Shanghai East Hospital, Tongji University School of Medicine, 150 Jimo Road, Pudong New District, Shanghai 200120, China. **E-mail**: naliubrown@163.com

Supplementary materials

Supplementary Figures 1-8

Supplementary Tables 1-2

Supplementary Material and Methods

Supplemental Figure 1

A

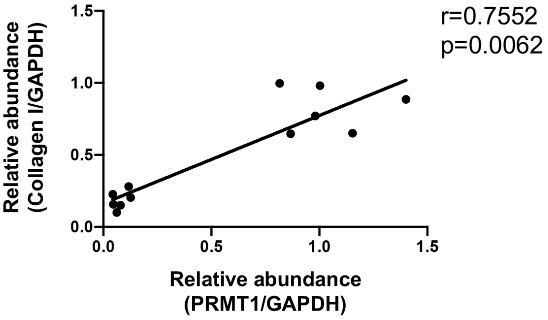

Supplementary Figure 1. The positive correlation between PRMT1 and Collagen I.

A) The correlation analysis between PRMT1 and Collagen I according to the immunoblotting results. 6 patients accepted catheter insertion operations, and 6 patients (duration of dialysis ≥ 26 months) accepted catheter removal operations because of ultrafiltration failure were enrolled in this study.

Supplemental Figure 2

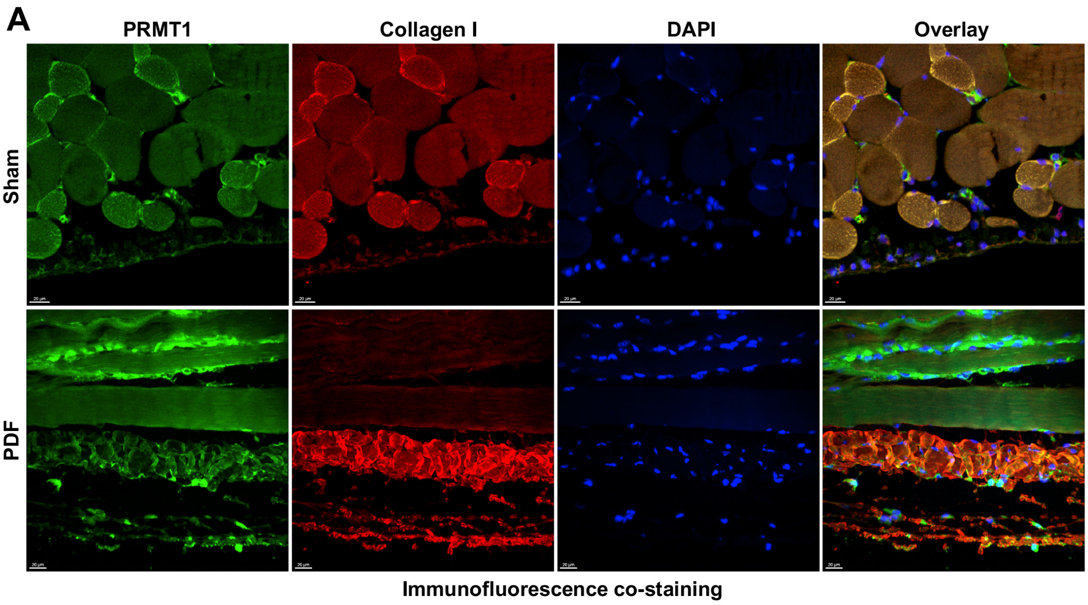

Supplementary Figure 2. PRMT1 overexpresses in Collagen I positive area.

A) Representative images of co-immunofluorescence staining of PRMT1 with Collagen I in the peritoneum of mice received HG-PDF or vehicle intraperitoneal injection for 28 days. Scale bars=20μm.

Supplemental Figure 3

A

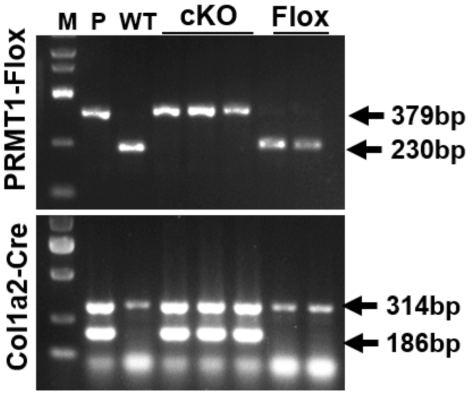

Supplementary Figure 3. Construction of PRMT1 conditionally knock-out mice.

A) The genotyping results of the transgene mice. In the image, P means positive control, WT means wildtype mice, cKO means PRMT1$^{fl/fl}$Col1a2 Cre^{+} mice, and Flox means PRMT1$^{fl/fl}$Col1a2 Cre^{-} mice.

Supplemental Figure 4

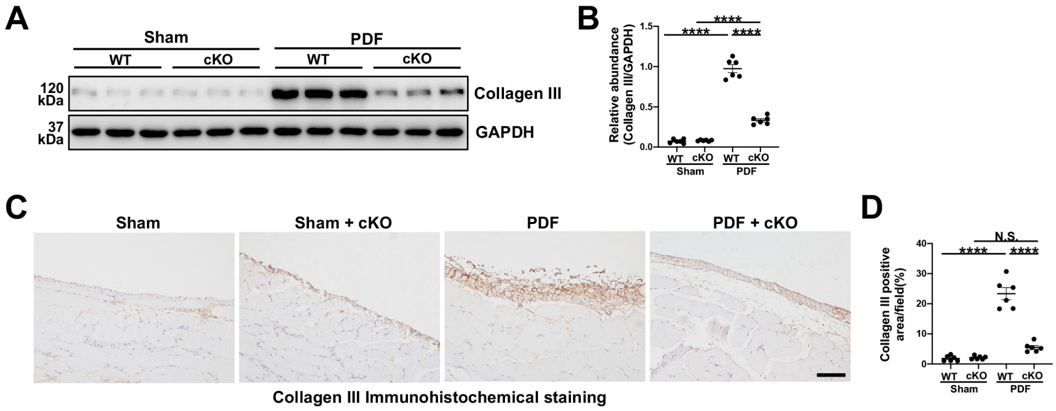

Supplementary Figure 4. Genetic depletion of PRMT1 reduces the matrix deposition in mice peritoneum.

A) The expression levels of Collagen III and GAPDH were determined by western blot, and **B)** the proteins were quantified by densitometry and normalized by GAPDH. **C)** Representative images of immunostaining of Collagen III in the peritoneum of wildtype or PRMT1-cKO mice received HG-PDF or vehicle intraperitoneal injection for 28 days. Scale bars=100μm. **D)** The positive area in peritoneum of different groups was quantified. Data were expressed as means ± SEM (n=6). N.S., no significant difference, *P<0.05, **P<0.01, ***P<0.001, ****P<0.0001.

Supplemental Figure 5

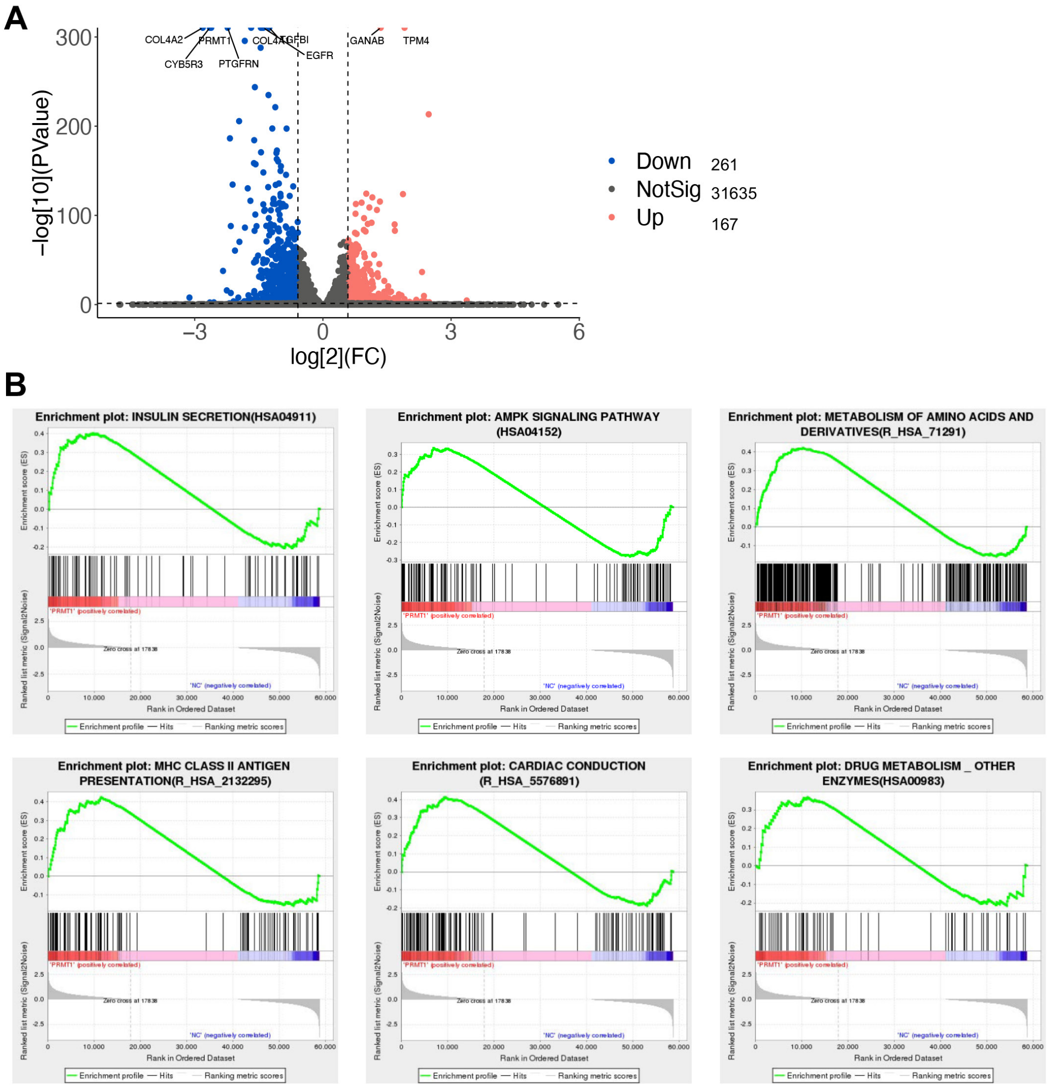

Supplementary Figure 5. Gene expression profile of PRMT1 knockdown human peritoneal mesothelial cells stimulated by TGF-β1 revealed by RNA sequencing analysis.

A) A volcano plot showing 428 differentially expressed genes. There were 261 upregulated genes and 167 downregulated genes compared with the control group. The heatmap shows the top differentially expressed genes (DEGs) between Vehicle and AMI-1-treated group HMrSV5 with TGF-β1 stimulation. Red signifies upregulated genes, and green indicates downregulated genes. Differential gene expression was displayed as a Z-score. **B)** Kyoto Encyclopedia of Genes and Genomes (KEGG) analysis shows the top enrichment pathways of DEGs.

Supplemental Figure 6

A

TGFBI|EGFR|COL4A1|HAVCR2|HSPB1|ATP13A2|ITGA6|PDGFB|JAG1|NET1|COL4A2|CDKN2B|SERPINE2|PHACTR3|SEMA7A|KCNA7|SLC12A8|RASSF10|SMURF2|LPCAT2|MOB3B|JUNB|HEG1|SERPINB7|NCS1|OXTR|RAB3B|LOX|F3|SLC4A7|MYOZ1|MAF|NCF2|ACKR2|FFAR1|ENTPD7|GPC6|DUSP7|SLCO2A1|PTGFRN|RASA1|PDCD1LG2|STK38L|TRHDE|C15orf48|KDELR2|CCNJ|REPS2|PIK3IP1|FEZ2|HACD2|EDN2|PTPN12|IQCJSCHIP1|CA2|SYT11|FNBP1L|RDH11|NTF4|ELMOD1|KIF5C|ACAN|SLC25A13|CMTM4|VMA21|PCDH1|REEP3|ELOVL7

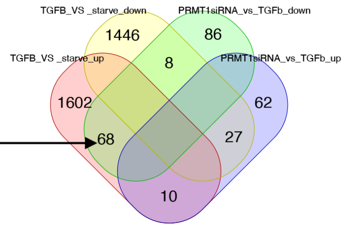

B

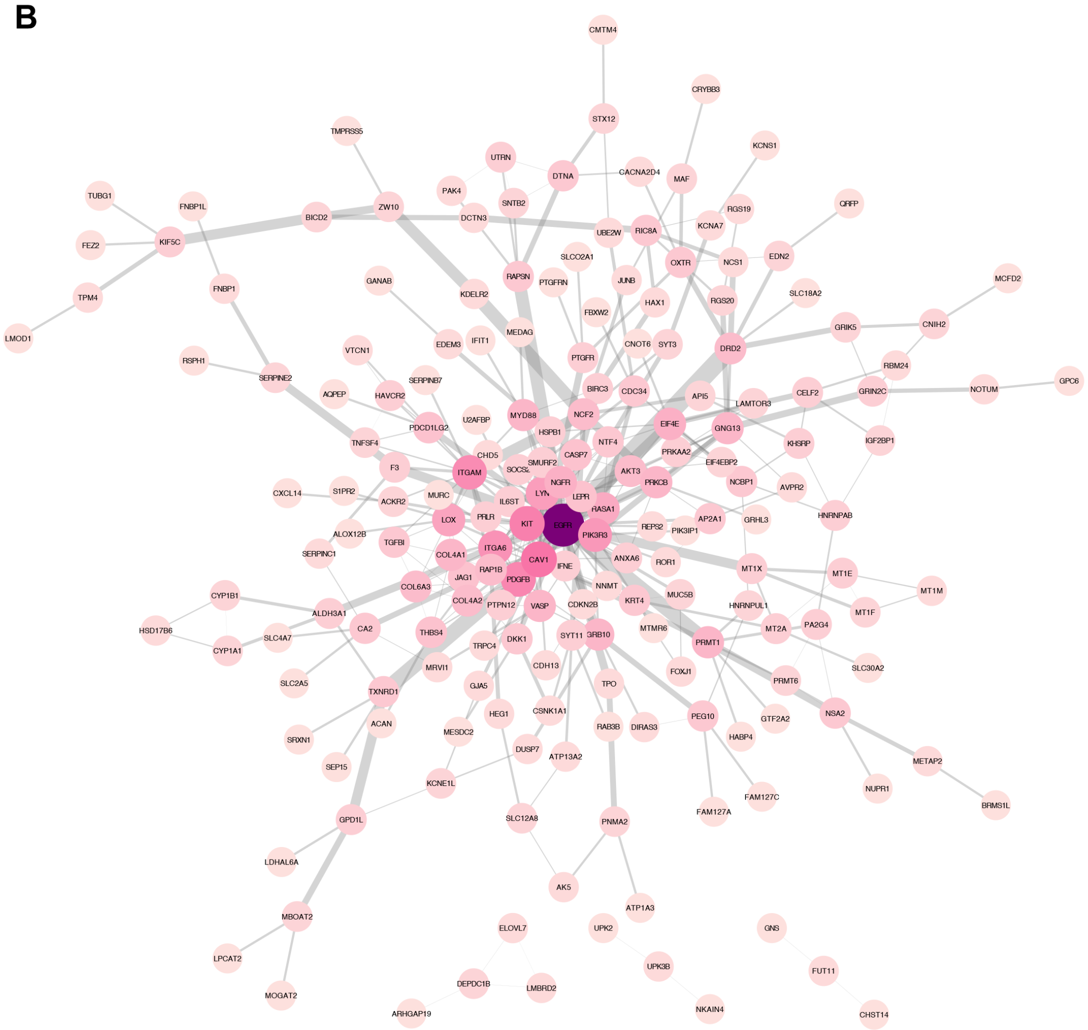

Supplementary Figure 6. PPI network and correlation analysis of the RNA sequencing.

A) Venn diagram shows the overlapping of DEGs in different groups of HMrSV5s; Upregulated genes in TGF-β1-treated (red), downregulated genes in TGF-β1-treated (yellow), upregulated genes in PRMT1-Knockdown group (blue), and downregulated genes in PRMT1-Knockdown group (green), respectively. **B)** Protein-protein interaction (PPI) network analysis of EGFR with associated DEGs obtained from the bulk RNA sequencing.

Supplemental Figure 7

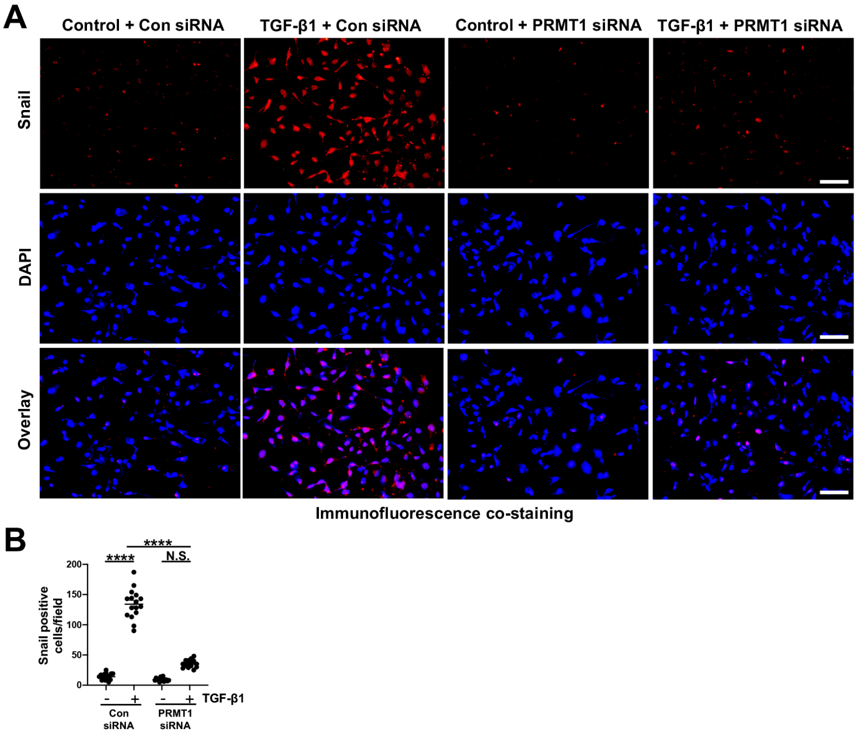

Supplementary Figure 7. Genetic depletion of PRMT1 suppresses the upregulation of Snail induced by TGF-β1 i*n vitro*.

A) Representative images of immunofluorescence staining of Snail in the NC or PRMT1 siRNA-treated HMrSV5s stimulated by TGF-β1 or Vehicle. Scale bars=100μm.

B) Quantization count of Snail positive cells in each field. Data were expressed as means ± SEM (n=4). N.S., no significant difference, *P<0.05, **P<0.01, ***P<0.001, ****P<0.0001.

Supplemental Figure 8

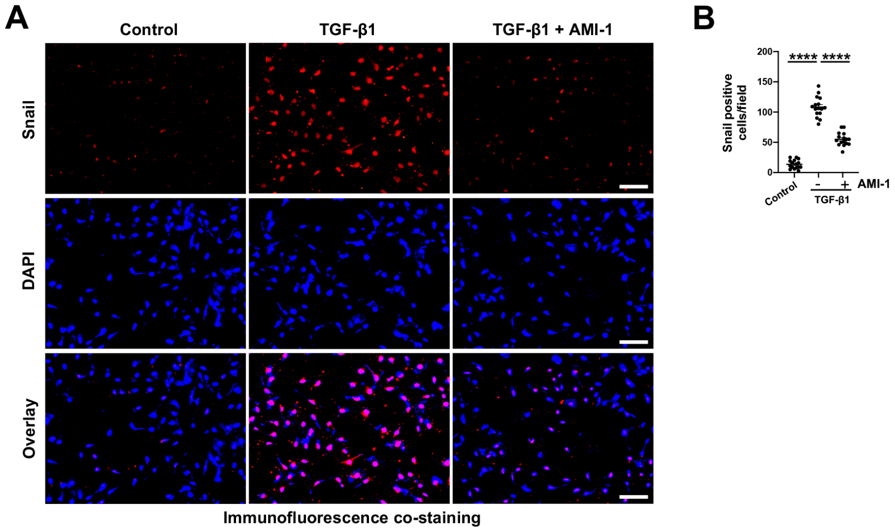

Supplementary Figure 8. Pharmacological inhibition of PRMT1 suppresses the upregulation of Snail induced by TGF-β1 i*n vitro*.

A) Representative images of immunofluorescence staining of Snail in the vehicle or AMI-1-treated HMrSV5s stimulated by TGF-β1 or Vehicle. Scale bars=100μm. **B)** Quantization count of Snail positive cells in each field. Data were expressed as means ± SEM (n=4). N.S., no significant difference, *P<0.05, **P<0.01, ***P<0.001, ****P<0.0001.

Supplementary Table 1. Clinical characteristic of the Non-PD and long-term PD patients

Variables	Non-PD patients (n=6)	Long-term PD patients (n=6)
PD time (months)	/	40.33±14.60
Age (years)	62.50±9.67	62.33±12.63
Male (%)	83.33%	66.67%
BMI (kg/m^2)	25.18±3.46	23.45±4.18
Drink (%)	33.33%	16.67%
Smoke (%)	50.00%	50.00%
Serum albumin (g/L)	34.52±6.83	30.80±9.45
TC (mmol/L)	4.28 (3.34-5.18)	3.50(2.77-6.04)
TG (mmol/L)	1.46 (1.02-2.42)	2.60(1.15-3.03)
HDL-C (mmol/L)	1.02 (0.81-1.28)	0.87(0.71-1.06)
LDL-C (mmol/L)	2.34 (1.92-3.42)	1.56(1.25-3.91)
Cr (μmol/L)	647 (364.25-896.25)	637(270.25-1056.25)
BUN (mmol/L)	25.22 (20.63-32.47)	15.28(10.90-20.70)
Sodium (mmol/L)	140.83±1.47	137.17±2.40
Potassium (mmol/L)	3.90±0.42	4.04±0.89
Calcium (mmol/L)	2.02±0.14	2.38±0.46
Hypertension (%)	83.33%	33.33%
Diabetes mellitus (%)	66.67%	66.67%
ACEI/ARB (%)	50.00%	33.33%
CCB (%)	83.33%	50.00%
Lipid-lowering drugs (%)	83.33%	50.00%
Insulin (%)	66.67%	66.67%

The continuous variables are reported as means ± SD or median and interquartile range (Me, IQR) according to the distribution. and categorical variables are presented as percentages. Abbreviations: BMI: body mass index; TC: total cholesterol; TG: triglyceride; HDL-C: high lipoprotein cholesterol; LDL-C: low density lipoprotein cholesterol; Cr: creatinine; BUN: blood urea nitrogen; ACEI/ARB: angiotensin converting inhibitor/ angiotensin receptor blocker; CCB: calcium channel blocker.

Supplementary Table 2. Clinical characteristic of the PD patients

PD Time / Variables	≤ 1 month (n=16)	1-12 months (n=22)	12-24 months (n=13)	24-36 months (n=12)	＞ 36 months (n=17)
PD time (months)	1.0 (0.0-1.0)	6.5 (3.8-8.3)	16.0 (14.0-20.0)	29.0 (26.0-33.5)	47.0 (43.5-59.5)
Age (years)	62.13±11.08	66.82±10.48	62.31±13.98	66.83±13.91	62.47±15.66
Male (%)	56.25%	50.00%	76.92%	83.33%	58.82%
BMI (kg/m^2)	24.61±3.66	23.36±2.89	23.91±2.64	25.48±4.99	26.48±3.26
Drink (%)	6.25%	9.10%	7.69%	16.67%	5.88%
Smoke (%)	31.25%	22.72%	30.77%	25.00%	5.88%
Serum albumin (g/L)	31.53±3.64	30.72±4.68	31.95±4.24	33.13±3.84	34.87±4.14
TC (mmol/L)	4.15(3.51-5.99)	4.44(3.73-5.34)	3.19(2.78-4.03)	3.50(3.14-4.24)	3.71(2.97-4.91)
TG (mmol/L)	2.19(1.49-3.11)	1.60(1.14-2.62)	1.38(1.01-2.83)	1.34(1.11-1.67)	2.96(1.50-3.87)
HDL-C (mmol/L)	0.92(0.82-1.11)	1.04(0.86-1.16)	0.94(0.65-1.14)	0.97(0.72-1.25)	0.80(0.70-1.10)
LDL-C (mmol/L)	2.51(1.47-3.88)	2.60(2.19-3.73)	1.93(1.21-2.24)	2.12(1.70-2.65)	1.98(1.49-2.53)
Cr (μmol/L)	644(466.2-858.3)	672.5(546-831.5)	858.0(667-1072.0)	876(729.5-1082.5)	912（818.5-1130.5）
BUN (mmol/L)	15.5(12.2-22.5)	17.3(13.6-20.5)	14.6(12.2-17.6)	19.9(13.6-22.0)	17.1(15.0-20.9)
Sodium (mmol/L)	139.29±2.55	141.29±2.14	140.29±3.00	140.04±2.34	140.30±2.79
Potassium (mmol/L)	3.86±0.70	3.63±0.50	3.90±0.50	3.60±0.51	3.88±0.66
Calcium (mmol/L)	2.24±0.29	2.23±0.22	2.30±0.19	2.28±0.25	2.43±0.18
Hypertension (%)	87.50%	72.72%	46.15%	66.67%	76.47%
Diabetes mellitus (%)	43.75%	54.54%	61.54%	66.67%	58.82%
ACEI/ARB (%)	68.75%	68.18%	69.23%	66.67%	41.18%
CCB (%)	100.00%	100.00%	100.00%	91.67%	52.94%
Lipid-lowering drugs(%)	25.00%	36.36%	38.46%	25.00%	47.06%
Insulin (%)	25.00%	50.00%	46.15%	66.67%	58.82%

The continuous variables are reported as means ± SD or median and interquartile range (Me, IQR) according to the distribution. Categorical variables are presented as percentages. Abbreviations: BMI: body mass index; TC: total cholesterol; TG: triglyceride; HDL-C: high lipoprotein cholesterol; LDL-C: low density lipoprotein cholesterol; Cr: creatinine; BUN: blood urea nitrogen; ACEI/ARB: angiotensin converting inhibitor/ angiotensin receptor blocker; CCB: calcium channel blocker.

Supplementary Material and Methods

Antibodies and Materials

AMI-1(S7884) was purchased from Selleckchem (Houston, TX, Unisted States). The primary antibodies to EGFR (#4267), p-EGFR(#3777), AKT (#9272), p-AKT (#4060), Snail (#3879), STAT3 (#9139), p-STAT3 (Tyr705) (#9138), E-cadherin (#14472), ERK1/2 (#4695), and p-ERK1/2 (Thr202/Tyr204) (#9101) were purchased from Cell Signaling Technology (Danvers, MA, United States). Antibodies to Fibronectin (ab2413), MMP2 (ab37150), and MMP9 (ab38898) were purchased from Abcam (Cambridge, MA, United States). Antibodies to GAPDH (sc-32233), Collagen I (sc-28654), and PRMT1 (sc-59648) were purchased from Santa Cruz Biotechnology (San Diego, CA, United States). α-SMA (A2547) was purchased from Sigma-Aldrich (St. Louis, MO, United States). Antibody to H4R3me2a (DF6954) was purchased from Affinity (Affinity Biosciences, United States). Collagen III (GB11023) was purchased from Servicebio (Wuhan, China).

The Use of clinical samples in the study

The peritoneum samples of PD patients were obtained from surgical tissues and conducted immunohistochemical/immunofluorescence staining and western blot. These patients were divided into 2 groups (Supplementary Table 1).

The PD effluents of patients with diverse PD duration time from 3 PD centers were collected when the patients conducted regular peritoneal equilibration test (PET). The supernatant is detected by immunoblotting assay and ELISA kits after high-speed centrifugation. The inclusion criterion is the continuous ambulatory peritoneal dialysis (CAPD) patients who without active peritonitis, severe heart failure, unstable angina, active liver disease, trauma or operation within the past 6 months.

Genotyping of the mice

The genomic DNA isolated from mice tail biopsies was analyzed by PCR to detect PRMT1 Wild type allele (230 bp), Mutant allele (379bp), Col1a2 Cre Transgene allele (186bp), and Col1a2 Cre Internal positive control (314bp).

The Genotyping primers for PRMT1 allele were 5′-TTTGCATGGGGATCTTT GGTGACT-3′ (Forward[F]), and 5′-GTAGAGCGGCAGGCCTGTGGTAAG- 3′(R e-verse[R]). The Genotyping primers for Col1a2 Cre allele were 5′-TTTGCATG GGGATCTTTGGTGACT-3′ (F), and 5′-CATGTCCATCAGGTTCTTGC-3′(R). Th e genotyping primers for Col1a2 internal positive allele were 5′-TGAAAAAGT CCACTAATTAAAACCA-3′ (F), and 5′-CTAACAACCCTTTCTCTCAAGGT-3′ (R).

Murine PF model

HG-PDF model: We randomly divided mice that weighed 20-25g into groups as followed: (a) WT-Sham (n=6): wild type mice injected with saline daily for 28 days; (b) cKO-Sham (n=6): PRMT1 cKO mice injected with saline daily for 28 consecutive days; (c) WT-PDF (n=6): wild type mice injected with 4.25% HG-PDF (100ml/kg) for 28 consecutive days; (d) cKO-PDF (n=6): PRMT1 cKO mice injected with 4.25% HG-PDF (100ml/kg) daily for 28 consecutive days. The mice were euthanatized on Day 28.

To investigate the effects of pharmacological inhibition of PRMT1 in the PF, we used AMI-1 in mice. The C57BL/6 mice were divided into the following groups randomly: (a) Sham (n=6): mice with intraperitoneal injection of vehicle and saline daily for 28 days; (b) Sham+AMI-1 (n=6): mice with intraperitoneal injection of AMI-1 (2mg/kg) and saline daily for 28 days; (c) PDF (n=6): mice with intraperitoneal injection of vehicle and 4.25% HG-PDF (100ml/kg) daily for 28 days; (d) PDF+AMI-1 (n=6): mice with intraperitoneal injection of AMI-1 (2mg/kg) and 4.25% HG-PDF (100ml/kg) daily for 28 days. At the end of 28 days, all mice were euthanatized by dislocation of the cervical vertebra. The peritoneum tissues and blood were collected from each mouse for further experiments. The 4.25% HG-PDF was obtained from Baxter Healthcare (Guangzhou, PR China).

Cell culture and treatments

HMrSV5s were cultured in Dulbecco's modified Eagle's medium with F12 (DMEM,

Biological Industries, Israel) containing 10% fetal bovine serum (FBS, Biological Industries, Israel), 1% penicillin, and streptomycin. The cells were kept in an atmosphere of 5% CO_2, and 95% air at 37°C. Before examining the mechanisms underlying *in vitro*, we starved HMrSVs for 24 hours with DMEM/F12 containing 0.5% FBS and then exposed to TGF-β1 (R&D, United States) with different concentrations (0, 1, 5, 10 ng/ml) or different treatment time (0, 12, 24, 36 hours). To verify the function and mechanisms of PRMT1 in PF, we used AMI-1 (5 μM) to treat HMrSV5 with stimulation of TGF-β1 (10 ng/ml) or vehicle for 36 hours. Then, cells were harvested for further experiments. All the in vitro experiments were repeated at least three times.

Enzyme-linked immunosorbent assay (ELISA) analysis

TGF-β1, MMP2, CA125, VEGF, and PRMT1 enzyme-linked immunosorbent assay (ELISA) kits were purchased from Cloud-clone Corp. (Wuhan, China). ELISA detection of proteins in mice peritoneum was performed following the manufacturer's instructions.

Western Blotting

Immunoblot analysis of peritoneum tissue samples or cell lysates was conducted following the manufacturer's instructions. The cells and tissues were lysed in RIPA buffer with protease inhibitor cocktail and phosphatase inhibitors. The protein concentrations of lysates were next quantified by BCA. Equal quality protein was resolved by using SDS-PAGE gel, transferred to PVDF membranes. The membranes then were incubated with corresponding primary antibodies overnight at 4°C. The PVDF membrane then were incubated with secondary antibodies next day for 1 hour at room temperature. Densitometry analysis of immunoblot results was conducted by using ImageJ software.

Immunoprecipitation (IP)

HMrSV5s with different treatments were seeded in culture dishes. Proteins were

extracted from those cells using lysis buffer and centrifuged at 10,000 ×g for 10 min at 4 °C. The cell lysates were then pre-cleared at 4 °C for 1 hour. Subsequently, adequate primary antibody or isotype IgG (Cell Signaling Technology, USA) was added to the cleared cell extracts and incubated at 4 °C overnight. Protein A/G PLUS-Agarose (Santa Cruz Biotechnology, USA) was added to the cell extracts and incubated at 4 °C for 1 hour. Then the beads were washed with buffer. Finally, the beads-bound proteins were eluted by adding 1X loading buffer and heated at 95 °C for 5 minutes for analysis.

Cleavage Under Targets & Release Using Nuclease (CUT&RUN) Assay

CUT&RUN assay (Cell Signaling Technology, USA) followed the manufacturer's instructions. Briefly, cells were cross-linked with 0.1% formaldehyde for 2 min at room temperature (RT) and then terminated. The chromatin was immunoprecipitated with a specific H4R3me2a antibody. DNA purification using phenol/chloroform extraction and ethanol precipitation. Analyze quantitative PCR results using the software provided with the real-time PCR machine detected using EGFR primers. The data was presented as a percentage relative to the input DNA. The primers for EGFR used in this study were listed as follows: 5′-ACCCATATGTACCATCGATGTC-3' (F) and 5'-GAATTCGATGATCAACTCACGG-3'(R).

Morphologic Studies of Peritoneum

The fixed peritoneum tissues were embedded in paraffin, cut into 4-μm-thick sections, and sectioned on slides. The slides were stained with Masson's trichrome staining and Sirius red staining to evaluate the degree of fibrosis and collagen deposits. The staining was performed according to the protocol provided by the supplier (Servicebio, Wuhan, China). The positive area of staining was quantitatively measured using Image Pro-Plus software (Media-Cybernetics, Silver Spring, MD, United States) by drawing a line around the perimeter of a positive staining area, and the ratio to each microscopic field was calculated and graphed. Slides were captured with a Nikon Eclipse 80i microscope with a digital camera (DS-Ri1, Nikon, Shanghai, China).

Immunohistochemical and immunofluorescence staining

Immunohistochemical and immunofluorescence staining in tissue were carried out according to the manufacturer's instructions. Sections were rehydrated and incubated with primary antibodies and then secondary antibodies (Beyotime, Shanghai, China). The slides for immunofluorescence staining were further stained with DAPI (1 μg/mL, Beyotime) and embedded with antifade mountant reagent (Invitrogen). Slides were captured with a Nikon Eclipse 80i microscope with a digital camera (DS-Ri1, Nikon, Shanghai, China).

For staining in cells, the HMrSV5s with different treatments were fixed with 4% paraformaldehyde for 20 min at room temperature. Cells were then incubated in a blocking buffer (5% goat serum in PBS) for 1 h at room temperature, followed by incubation with primary antibodies at 4 °C overnight. Cells were washed thrice in PBS and then incubated with secondary antibodies for 1 hour at room temperature. Finally, the nuclei were stained with DAPI (1 μg/mL, Abcam) for 15 min at room temperature before visualization. Cells were observed with ZEISS Digital Camera for Fluorescence Microscopy.

Statistical analysis

All the experiments were conducted at least three times. Data depicted in graphs are expressed as means ± S.E.M. for each group. Student's t-test was employed for comparisons between two groups and one-way analysis of variance (ANOVA) followed by Tukey's post-test for multiple comparisons was used for groups of three or more. All tests were two-tailed. The *P*-value less than 0.05 was considered statistically significant and was marked in each graph. $P<0.05$ was considered significant. The statistical analyses were conducted by using IBM SPSS Statistics 20.0 (Version X; IBM, Armonk, NY, United States).

Bulk RNA-sequencing

RNA integrity was assessed using the RNA Nano 6000 Assay Kit of the Bioanalyzer

2100 system (Agilent Technologies, CA, USA). Total RNA was used as input material for the RNA sample preparations. Briefly, mRNA was purified from total RNA using poly-T oligo-attached magnetic beads. Fragmentation was performed using divalent cations under elevated temperature in the First Strand Synthesis Reaction Buffer(5X). First-strand cDNA was synthesized using random hexamer primer and M-MuLV Reverse Transcriptase, then use RNase H to degrade the RNA. Second-strand cDNA synthesis was subsequently performed using DNA Polymerase I and dNTP. The remaining overhangs were converted into blunt ends via exonuclease/polymerase activities. After the adenylation of 3' ends of DNA fragments, an Adaptor with a hairpin loop structure was ligated to prepare for hybridization. To select cDNA fragments of preferentially 370~420 bp in length, the library fragments were purified with the AMPure XP system (Beckman Coulter, Beverly, USA). Then, PCR was performed with Phusion High-Fidelity DNA polymerase, Universal PCR primers, and Index (X) Primer. Finally, PCR products were purified (AMPure XP system), and library quality was assessed using the Agilent Bioanalyzer 2100 system.

Clustering and sequencing

The index-coded samples were clustered on a cBot Cluster Generation System using TruSeq PE Cluster Kit v3-cBot-HS (Illumia) according to the manufacturer's instructions. After cluster generation, the library preparations were sequenced on an Illumina Novaseq platform, and 150 bp paired-end reads were generated.

Raw data (raw reads) of fastq format were firstly processed through in-house perl scripts. Reference genome and gene model annotation files were downloaded from genome website directly. Index of the reference genome was built using Hisat2 v2.0.5 and paired-end clean reads were aligned to the reference genome using Hisat2 v2.0.5. feature Counts v1.5.0-p3 was used to count the reads numbers mapped to each gene. And then FPKM of each gene was calculated based on the gene length and reads count mapped to this gene. Differential expression analysis of two conditions/groups (two biological replicates per condition) was performed using the DESeq2 R package (1.20.0). Gene Ontology (GO) enrichment analysis of differentially expressed genes

was implemented by the cluster Profiler R package, in which gene length bias was corrected. PPI analysis of differentially expressed genes was based on the STRING database, which known and predicted Protein-Protein Interactions.
